# A survey of molecular diversity and population genetic structure in North American clearwing moths (Lepidoptera: Sesiidae) using cytochrome *c* oxidase I

**DOI:** 10.1371/journal.pone.0202281

**Published:** 2018-08-22

**Authors:** Linda A. Lait, Paul D. N. Hebert

**Affiliations:** Centre for Biodiversity Genomics, University of Guelph, Guelph, Ontario, Canada; National Cheng Kung University, TAIWAN

## Abstract

The phylogeographic structure of insect species in North America is poorly understood. The moth family Sesiidae (Lepidoptera) contains many economically important pests of agriculture and forestry, as well as beneficial species used in biological control. Despite their significance, this study constitutes the first broad-ranging population genetic study on North American sesiids. It probes the population structure of eight species of sesiid moths based on sequence variation in cytochrome *c* oxidase I (N = 191). Haplotype diversity levels were high in seven of the eight species, while nucleotide diversity varied considerably. Patterns ranged from limited structure and a starburst pattern in the raspberry crown borer *Pennisetia marginata* to highly geographically structured populations in the peachtree borer *Synanthedon exitiosa* and the maple callus borer *Synanthedon acerni*. These varied patterns suggest differing evolutionary histories and dispersal abilities. By elucidating population genetic structure and barriers to dispersal we can begin to devise conservation and management plans.

## Introduction

Studies of population genetic structure can reveal where a species persisted through time, how it colonised new regions, and whether current populations experience ongoing gene flow. The evolutionary histories of North American species have been heavily influenced by the Pleistocene glaciations [1–3], with the most recent glaciation, the Wisconsin glaciation, resulting in alteration of habitat distribution [4, 5]. As a result, many species had to persist in ice-free refugia, primarily located south of the ice sheets and in a large ice-free region in Beringia, although recent studies have shown that periglacial regions on both coasts and in the Arctic may also have supported taxa [1, 6–8]. Contemporary dispersal capabilities are also reflected in the population genetic structure of a species. Highly vagile species such as birds, large mammals, and marine fishes often show limited geographic patterns [9–12], whereas species with restricted ranges, those affected by either physical or non-physical barriers, and those with poor dispersal capabilities often have significant population genetic structure (e.g., [12]). Such insights into evolutionary history can aid management efforts, be it a recovery plan for a species of conservation concern, or control programs for invasive and pest species. For example, the identification of isolated populations can help to target conservation efforts. In addition, a deeper understanding of how a species moves between areas can help to devise strategies to limit its capacity to invade new regions.

Although insects comprise nearly two thirds of animal diversity [13, 14], they are underrepresented in the population genetics literature—particularly among studies of eastern North American taxa—relative to their richness and abundance. Studies have revealed varying phylogeographic patterns: limited genetic structure was found in the endangered burying beetle *Nicrophorus americanus* [15], the cabbage looper *Trichoplusia ni* [16], and the monarch butterfly *Danaus plexippus* [17]; whereas significant structure and distinct lineages have been identified in the wheat stem sawfly *Cephus cinctus* [18], the walnut twig beetle *Pityophthorus juglandis* [19], and the yellow fever mosquito *Aedes aegypti* [20]. Several studies have examined butterfly species: highly vagile and migratory species exhibit high levels of gene flow and little regional differentiation [17, 21, 22], while alpine species show significant differences between populations on different mountain ranges [23, 24]. The disparate findings among these taxa suggest that further study is necessary.

The Sesiidae (Lepidoptera), or clearwing moths, are a small, broadly distributed family of moths found globally [25]. Its most recent global checklist includes 1,452 species in 160 genera [26]. The North American fauna includes 133–135 species assigned to 20 genera [26, 27] with three recent introductions from the Palaearctic (*Sesia apiformis*, *Synanthedon myopaeformis*, and *Synanthedon tipuliformis*). In addition, two species have a natural Holarctic distribution and 41 species are shared with the Neotropics [26, 28]. The larvae of sesiids are primarily host specialists which bore into the roots and stems of a single genus or family of trees, shrubs, vines, or herbs [25]. As a result, many sesiids are pests of agricultural, forestry, or ornamental plants. For example, the peachtree borer *Synanthedon exitiosa* and the dogwood borer *Synanthedon scitula* cause substantial damage in peach and apple orchards, respectively [29–31], while the maple callus borer *Synanthedon acerni* and the ash borer *Podosesia syringae* cause significant damage to hardwood forests [30, 32–34].

Despite their importance as agricultural and forestry pests, and their corresponding use as biological control agents, there is little molecular data on sesiids. There is currently no comprehensive molecular phylogeny for this family, although two studies generated regional phylogenies using short fragments of the mitochondrial cytochrome oxidase I and II genes for 20 and 21 species from 10 and 12 genera, respectively [35, 36], while a single study has explored the population genetic structure of the sesiid *Synanthedon pictipes* revealing multiple genetic lineages within a small geographical area [37]. With over 6 million publicly available barcode records (www.boldsystems.org; [38]), and ~4.5 million insect records, there is a wealth of data available. The present study aims to increase our understanding of the population genetic structure of this group by making use of existing DNA barcode records to examine levels of genetic variation and both past and contemporary isolation in North American Sesiidae.

## Materials and methods

### Sequences

Sequences of the 658 bp barcode region of the mitochondrial cytochrome *c* oxidase I gene were downloaded from the Barcode of Life Database (BOLD) [38] in April 2018 for all 752 sesiids from Canada and the United States (see S1 Table). Locations were recorded by state or province. Sequences were aligned in MEGA v6 [39]. In order to confirm the monophyly of each species a phylogeny was constructed in Mr. Bayes v3.2 [40] using the generalised time reversible model with gamma-distributed rate variation and invariable sites (GTR+Γ+I). The analysis was run for 5 million runs with a 25% burn-in, standard deviation of split frequencies < 0.02, and final potential scale reduction factor (PSRF) > 0.9999. *Xanthocastnia evalthe* (Lepidoptera: Castniidae; GenBank Accession Number HM377853) was used as the outgroup.

### Genetic analyses

**Fig 1:**
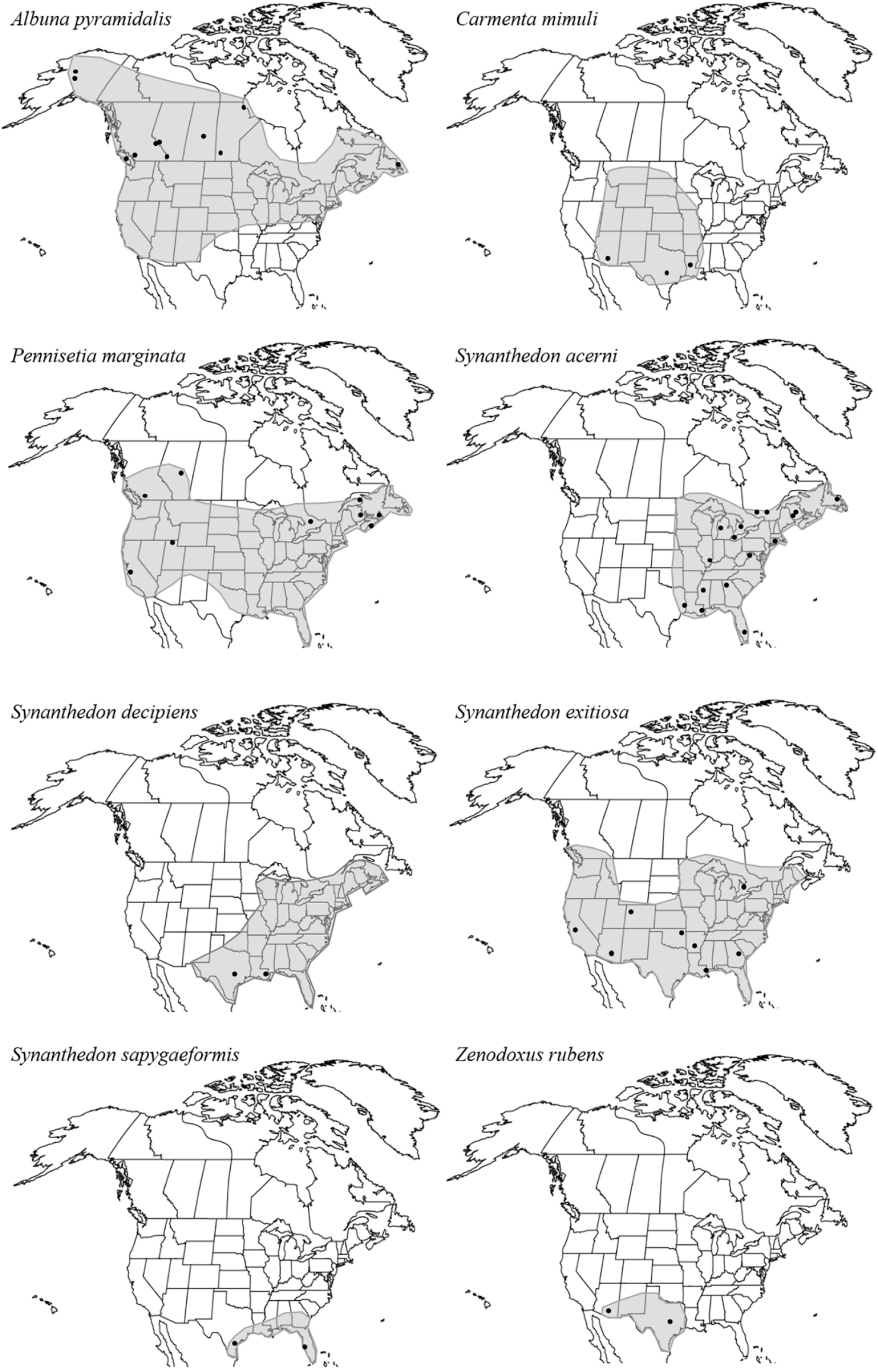
Approximate distributions (shaded) and sampling locations for eight North American sesiid species. The points represent sampled sites; multiple sites in the same region (e.g., ON) were grouped for subsequent analyses.

Eight species were selected for further intraspecific analyses (Fig 1): *Albuna pyramidalis*, *Carmenta mimuli*, *Pennisetia marginata*, *Synanthedon acerni*, *Synanthedon decipiens*, *Synanthedon exitiosa*, *Synanthedon sapygaeformis*, and *Zenodoxus rubens*. Haplotypes were assigned with TCS v1.21 [41] and confirmed by visual inspection. Haplotype (H_d_) and nucleotide (π, per site) diversity measures and neutrality tests (Tajima D and Fu’s Fs) [42, 43] were run in DnaSP v5.10 [44, 45]. To test for population structure, an analysis of molecular variance (AMOVA; 100,000 permutations) [46] and pairwise genetic differences (Φ_ST_; 100,000 permutations) for each species were calculated in Arlequin v3.5.2.2 [47]. The AMOVA allocated the genetic variation within and among sampling locations. For the pairwise comparisons, a modified false discovery rate correction (FDR) [48] was applied to correct for multiple tests. To test for evidence of population expansion a mismatch distribution analysis [49] was run in DnaSP v5.10 [44, 45].

In order to visualise the pattern of variation, a statistical parsimony network was constructed in TCS v1.21 [41] with a 95% connection limit. Bayesian clustering analysis was performed in BAPS v5.2 (Bayesian Analysis of Population Structure) [50] to assign specimens to clusters based on Bayes’ theorem with no *a priori* population information. Clustering analysis was run from K = 1 to 10 with the linked loci option [51].

## Results

### Samples

The 752 sesiid records downloaded included 662 specimens that could be assigned to one of 117 species either based on previous taxonomic identification or by membership in a Barcode Index Number (BIN) [52] assigned to a species. Of these 558 samples representing 100 species had full COI barcode sequences (≥ 540 bp; S1 Table).

### Bayesian analyses

**Fig 2:**
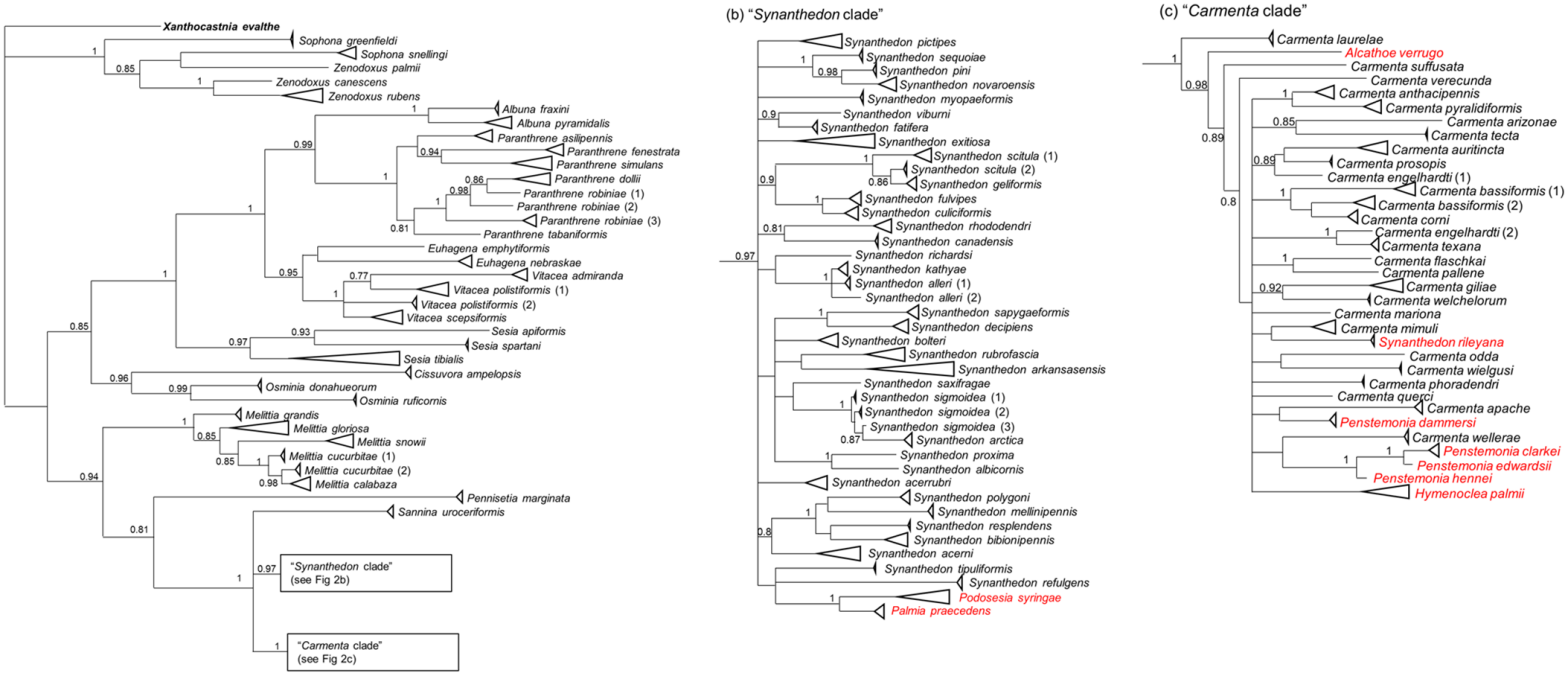
Bayesian analysis based on 558 COI barcodes among 100 North American sesiid species. The triangles represent multiple specimens from the same species, with the length of the triangle representing the amount of sequence variation. Posterior probabilities >0.8 are given. The full uncollapsed tree is available upon request.

The Bayesian network supported the monophyly of most species, including the eight species selected for further analyses (Fig 2). Most genera formed monophyletic groups, with the exceptions that *Sophona* and *Zenodoxus* were paraphyletic while *Palmia* and *Podesesia* fell within a larger *Synanthedon* clade. Finally, *Alcathoe*, *Hymenoclea*, *Penstemonia*, and *Synanthedon rileyana* all fell within a larger *Carmenta* clade. Many of these exceptions have been noted before [35, 36] and are likely due to 1) the recent separation of sister species, and/or 2) the generic misplacement of *Synanthedon rileyana*. Further work with additional specimens and markers is needed to increase the resolution of the phylogenetic relationships within this family.

### Population structure

Eight species were selected for further analysis, with coverage ranging from eight to 47 specimens and from two to 13 sampling locations (Table 1). Levels of diversity varied considerably among taxa, ranging from two variable sites in *Pennisetia marginata* to 54 variable sites in *Synanthedon exitiosa*. Haplotype diversity ranged from 0.25 to 0.98, while nucleotide diversity ranged from 0.008 to 0.027 (Table 1). None of the neutrality tests showed significant results.

**Table 1:** Sample size (N), number of sampling locations (Loc), variable sites (VS), number of haplotypes (h), haplotype diversity (H_d_), nucleotide diversity (π), and overall Φ_ST_ for eight North American sesiid species.

Species	N	Loc	VS	h	H_d_	π	Φ_ST_^1^
*Albuna pyramidalis*	28	6	40	22	0.98	0.0132	0.234**
*Carmenta mimuli*	17	3	27	10	0.84	0.0112	0.158
*Pennisetia marginata*	36	8	2	3	0.25	0.0079	0.429**
*Synanthedon acerni*	47	13	45	22	0.92	0.0134	0.881***
*Synanthedon decipiens*	10	2	16	8	0.96	0.0078	0.480**
*Synanthedon exitiosa*	27	8	54	18	0.96	0.0271	0.696***
*Synanthedon sapygaeformis*	17	2	24	13	0.96	0.0077	n/a
*Zenodoxus rubens*	8	2	34	7	0.96	0.0224	0.516

^1^ *p<0.05, **p<0.01, ***p<0.001

**Fig 3:**
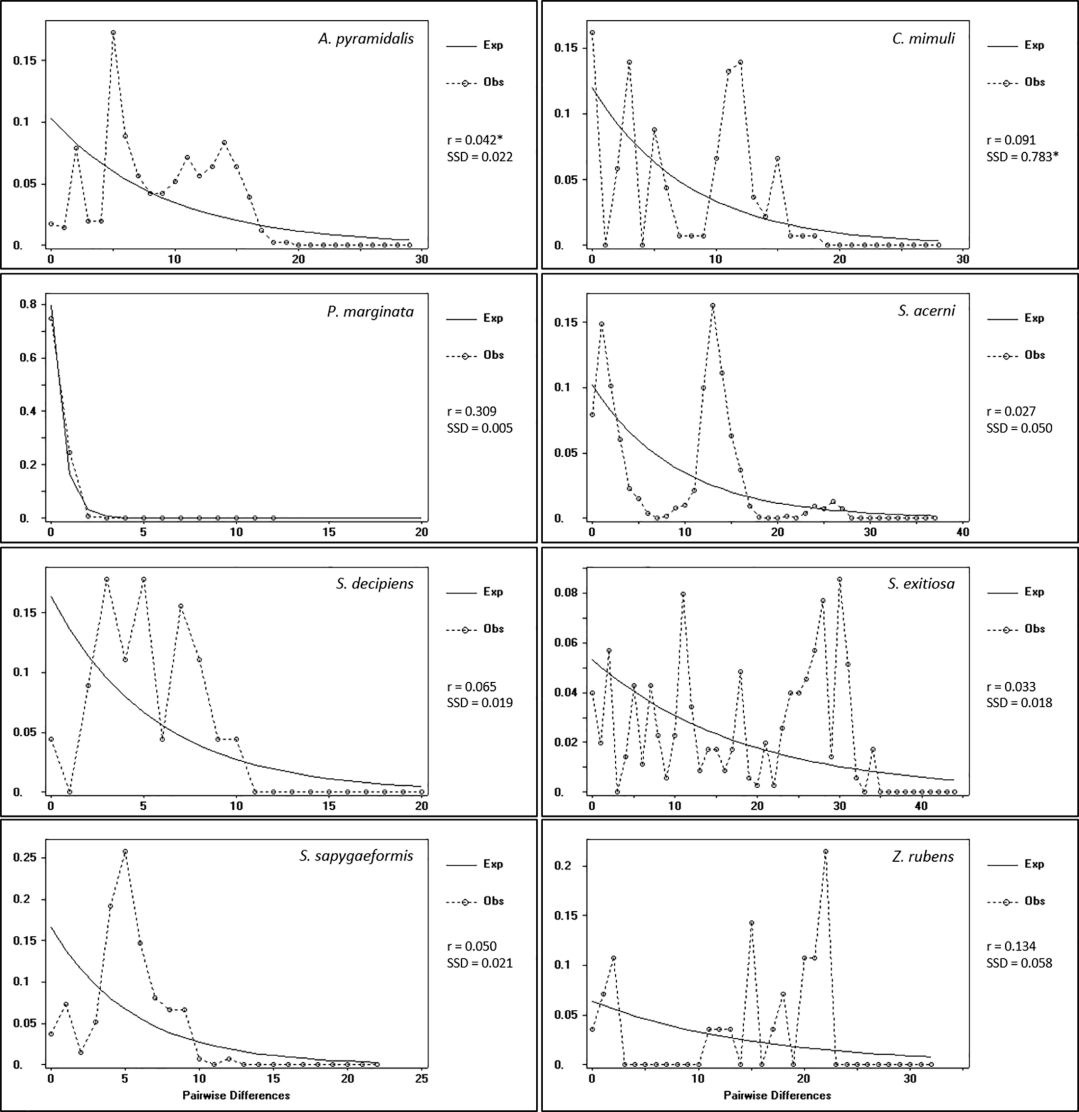
Mismatch distribution for eight North American sesiidae species. Harpending’s raggedness index (r) and the sum of squared deviations (SSD) are given. Significant values are depicted with a ‘*’.

The overall AMOVAs produced Φ_ST_ values ranging from 0.16 to 0.88. P-values were significant for *Albuna pyramidalis*, *Pennisetia marginata*, *Synanthedon acerni*, *S*. *decipiens*, and *S*. *exitiosa*, but not for *Carmenta mimuli* or *Zenodoxus rubens* (Table 1). As *Synanthedon sapygaeformis* was primarily represented by a single population, neither overall nor pairwise Φ_ST_ values were calculated for this species. Pairwise mismatch distributions showed relatively low but non-significant Harpending’s raggedness index values, with that of *P*. *marginata* an order of magnitude higher than the others (Fig 3). There was a multimodal distribution in all but *Pennisetia marginata* (Fig 3), likely the result of population structure within the species rather than genuinely stable populations. This is supported by the lack of significance in all but two of the indices.

**Fig 4:**
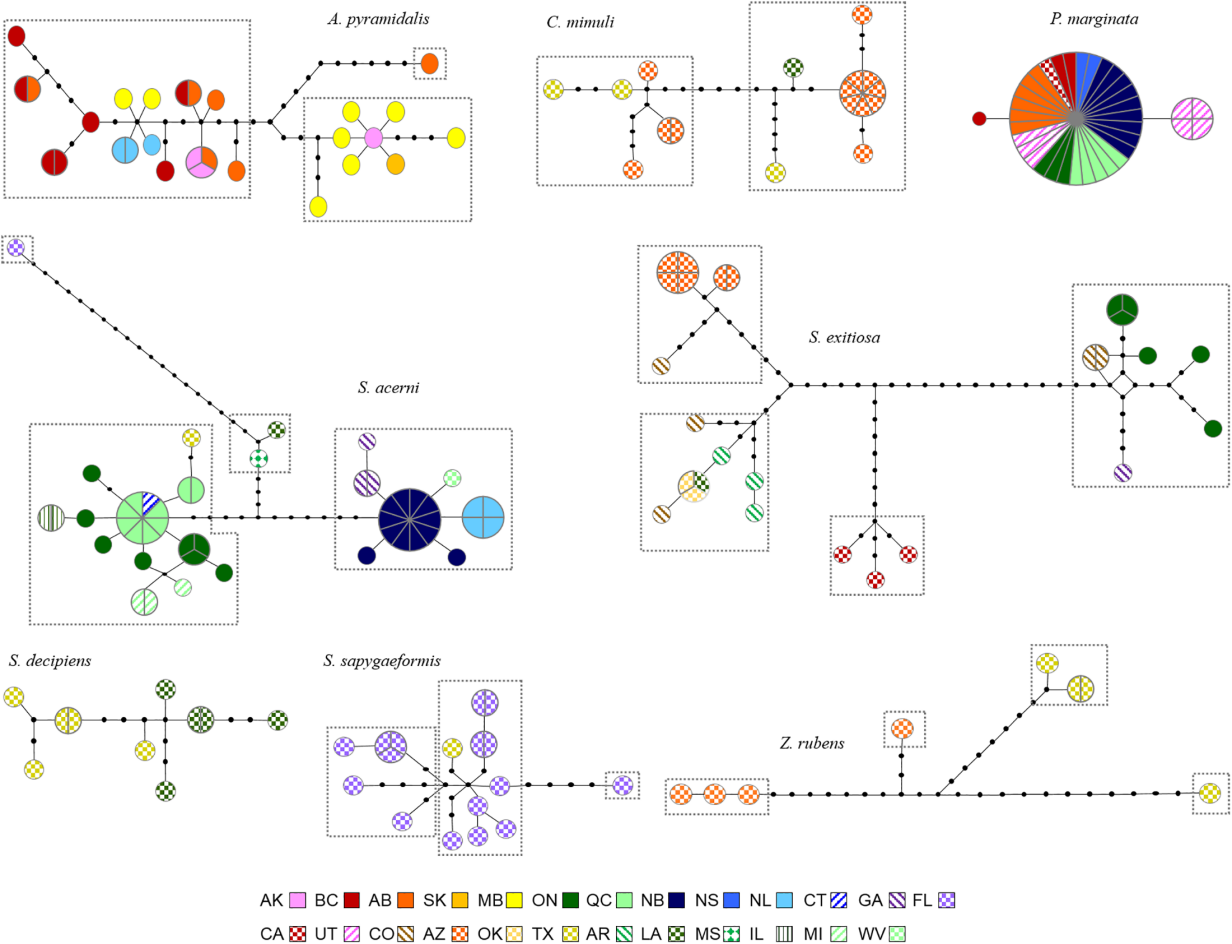
Statistical parsimony networks for eight North American sesiidae species. Each haplotype is represented by a circle where slices/sections represent multiple individuals with the same haplotype, and inferred haplotypes are shown by black dots. Circles/slices are colour-coded by sampling location, and the dashed boxes represent clusters identified in BAPS v5.2.

The eight species had very different patterns of variation (Table 2, Fig 4). *A*. *pyramidalis*, *S*. *acerni*, and *S*. *exitiosa* all possessed extensive variation (40, 45, and 54 variable sites, respectively), but *A*. *pyramidalis* had no significant population pairwise comparisons. By contrast, *S*. *acerni* and *S*. *exitiosa* had significant values for most comparisons with both showing a distinct east/west pattern (Table 2, Fig 4). Both the statistical parsimony network and the Bayesian clustering analyses supported the separation of samples into multiple groups (Fig 4), with BAPS identifying three, four, and four groups with probabilities of 0.943, 1.0, and 1.0, respectively. In *A*. *pyramidalis*, one group consisted of most Manitoba samples along with the sole Saskatchewan sample, while a second contained a single Alberta sample showing 14 substitutions from any other lineage, and the third group contained all other samples from both eastern and western locations. In *S*. *acerni*, samples from Atlantic Canada, West Virginia, and Georgia formed one group, while samples from Ontario, Quebec, Connecticut, Illinois, Michigan, and Texas formed a second, and a third southern group included one sample each from Louisiana and Mississippi, and the fourth group with a single sample from Florida that possessed 21 substitutions from any other, although it still grouped within *S*. *acerni* (Fig 4). In *S*. *exitiosa*, the groups roughly represented California, Arizona, the south (Oklahoma, Louisiana, and Arkansas), and the east (Ontario and Georgia); Colorado samples were found in all but the California group (Fig 4). *Carmenta mimuli* and *Zenodoxus rubens* showed high levels of variation between Arizona and Texas (Fig 4), although none of the population comparisons were significant (Table 2). In both cases Bayesian clustering identified multiple groups (two in *C*. *mimuli* and four in *Z*. *rubens*; probability = 0.996 and 1, respectively). *Synanthedon decipiens* showed significant differences between the two sampled locations (Table 2, Fig 4), reflecting the fact that they shared no haplotypes, but Bayesian clustering could not detect any significant groups. *Synanthedon sapygaeformis* showed a diverse network of closely related haplotypes sampled almost entirely from a single location (Fig 4); BAPS identified three groups (probability = 0.946). In contrast, *P*. *marginata* showed limited variation (two variable sites) with a single widespread haplotype found in 86.1% (31/36) of the samples with representation from both eastern and western locations (see Figs 1 and 4). Bayesian clustering could not identify any groups in *P*. *marginata*, although there was a single significant pairwise comparison due to the private haplotype present in Utah (Table 2).

**Table 2:** Population pairwise Φ_ST_ values for seven North American sesiid species. Significant comparisons after correction for multiple tests are shown in bold and shaded. Negative Φ_ST_ values are treated as 0.000.

*Albuna pyramidalis*							*Pennisetia marginata*					
	**sAB**	**BC/AB**	**sBC**	**nMB**	**NL**			**AB**	**BC**	**NB/NS**	**ON**	**QC**
**BC/AB**	0.136						**BC**	0.250				
**sBC**	0.328	0.017					**NB/NS**	0.000	0.441			
**nMB**	0.125	0.277	0.330				**ON**	0.000	0.000	0.000		
**NL**	0.376	0.298	0.379	0.337			**QC**	0.000	0.189	0.000	0.000	
**AK**	0.000	0.196	0.390	0.000	0.479		**UT**	0.472	0.333	**0.585**	0.344	0.439
*Synanthedon acerni*								*Synanthedon exitiosa*				
	**NB**	**NL**	**ON**	**QC**	**GA**	**MI**			**ON**	**AZ**	**STH**	**CA**
**NL**	**0.788**							**AZ**	**0.901**			
**ON**	**0.924**	**0.900**						**STH**	**0.849**	**0.804**		
**QC**	**0.972**	**0.979**	**0.177**					**CA**	**0.803**	**0.894**	**0.767**	
**GA**	**0.731**	0.884	**0.881**	**0.968**				**CO**	**0.389**	**0.477**	**0.277**	0.343
**MI**	**0.968**	0.967	**0.390**	**0.811**	0.938			STH = AR, LA, and OK				
**STH**	**0.884**	0.792	**0.592**	**0.741**	0.730	0.520						
STH = LA, MS, and TX												
*Carmenta mimuli*					*Synanthedon decipiens*					*Zenodoxus rubens*		
	**AZ**					**LA**					**AZ**	
**TX**	0.158				**TX**	**0.480**				**TX**	0.516	

## Discussion

The present study shows that North American sesiids exhibit a wide diversity of genetic variation and population genetic structure. The species are similar in size and dispersal ability, and are all specialists on a particular family or genus of host plant, although some target a single species while others feed on several [25]. While some of the variability in population genetic structure can be explained by differences in distribution, geographic coverage, and sample sizes, other patterns were unexpected.

### Albuna pyramidalis

The extensive haplotype variation in *Albuna pyramidalis* without significant population structure between eastern and western locations suggests a postglacial expansion from a single glacial refugium, at least in the northern part of its range. Similar patterns have been reported in many northern species of trees, birds, and mammals. For example, Graham and Burg [53] found that *Picoides villosus* exhibits little or no structure in the boreal and eastern portion of its range; similarly, Milá *et al*. [54] identified a single genetic lineage in *Dendroica coronata* across Canada and Alaska. In both cases it was proposed that northern locations derived from a single refugium, likely in the south. Other species possess a single lineage across eastern North America east of the Rocky Mountains [55–58], or across all of Canada [59, 60]. Samples from the southern portion of the range are required to test whether the species as a whole persisted in a single refugium, or whether there is additional structure in the south, particularly in the southwest, as seen in some of the other sesiid species.

The high level of variation in the species may be reflective of the extensive morphological variation observed in *A*. *pyramidalis*; the species has been noted for its highly variable colouration with five colour forms described [25]. While three of the colour forms may be present in the region sampled (nominate, *coloradensis*, and *montana*), the groupings found with BAPS did not correspond to the colour variations suggesting that the different forms may breed extensively or have only recently diverged. The two most distinctive forms, *rubescens* and *beutenmulleri*, are found only in Utah and Colorado and were not sampled in this study.

### Pennisetia marginata

The complete lack of population structure in *Pennisetia marginata* may reflect high levels of gene flow, a recent origin in North America, or a combination of these factors. High dispersal capability and gene flow among regions may occur due to a continuous distribution of wild and introduced populations of its host plant species (*Rubus*) [25]. While the suggested distribution of *P*. *marginata* is essentially disjunct, with only a few scattered records from the Great Plains, the species may traverse this central region without establishing large or long-term populations due to unfavourable conditions. Although extensive gene flow would explain haplotype sharing among geographically distant locations (i.e., British Columbia and Nova Scotia), it does not explain the limited variation despite a substantial sample size. A starburst pattern with a single common haplotype and a few others one or two mutations away has frequently been noted in fishes, birds, and mammals [61–65], and often indicates a recent rapid expansion following a bottleneck [66]. This is supported by the unimodal distribution in the mismatch analysis (Fig 3). The pattern could also be a result of an evolutionarily young species; Eichlin and Duckworth [25] noted that *P*. *marginata* is morphologically and behaviourally similar to the Palaearctic congeneric *P*. *hylaeiformis*, although consistent differences in male genitalia were able to distinguish the species. Examination of the cytochrome *c* oxidase I sequences supports the separation of these species with 3.2–4.0% divergences, suggesting that the divergence may have occurred relatively recently, possibly following a bottleneck.

### Synanthedon acerni

*Synanthedon acerni* exhibited clear geographic differences between “eastern” (NB, NL, WV, GA) and “western” (ON, QC, IL, TX) locations, with southern locations forming two additional groups (LA and MS; and FL). This pattern suggests their derivation from multiple glacial refugia, a physical barrier preventing gene flow, separation due to host specificity, or a combination of factors. As discussed above, the presence of multiple glacial refugia in North America is certain [1], and genetic differentiation between populations from the Atlantic and Gulf regions is common in species whose modern range spans these two regions [67, 68]. As well, several studies have shown that both the Mississippi River and Appalachian Mountains are often barriers to gene flow [69–71]. *S*. *acerni* is a specialist on *Acer* (Sapindaceae) species, particularly on red maple, *A*. *rubrum*, with which it shares its distribution, and silver maple, *A*. *saccharinum* [25]. Both species occur across eastern North America, with *A*. *rubrum* extending further east and *A*. *saccharinum* absent from Newfoundland [72]. Given this pattern it may suggest that the “eastern” lineage uses *A*. *rubrum* as its preferred host, while the “western” group is found on *A*. *saccharinum*. Studies of host species specificity could be used to test this further.

While three colour forms have been described for *S*. *acerni* [25], the eastern, western, and southern (LA and MS) groups all corresponded to the nominate form. The sample from Florida represented the colour form *buscki* which, given the high divergence between it and the other samples, may represent a separate species, or at the very least a form with a different host species. Additional work with nuclear markers and extensive sampling in the southeast region is required to confirm this.

### Synanthedon exitiosa

*Synanthedon exitiosa* showed clear geographic structure with four distinct lineages: Arizona, California, the southern United States, and the eastern United States and Canada. This may reflect its isolation in multiple glacial refugia with differences retained due to low gene flow among regions, varying host preferences leading to adaptations, or incipient speciation. Distinct population structure as a result of multiple glacial refugia has been observed in many plant and animal species, particularly in the west [54, 56, 67, 73–75]. The current distribution of *S*. *exitiosa* includes a number of putative refugial areas in the southern United States and the coastal Pacific Northwest [1], supporting the suggestion that it persisted in multiple Pleistocene refugia. The importance of host specificity on genetic isolation and incipient speciation has also been demonstrated in several herbivorous insect species, with changes in host preference leading to reproductive isolation [76, 77]. *S*. *exitiosa* feeds extensively on peach trees *Prunus persica* (Rosaceae), an introduction from Asia, as well as several other *Prunus* species, both native and introduced [25]. Many of its introduced hosts have narrow distributions in North America (e.g., *P*. *amygdalus* only occurs in California and Utah), while there are numerous native Rosaceae species, many of which have small ranges [72].

*S*. *exitiosa* also exhibits extensive morphological variation, with numerous colour forms described [25]. The four groups identified by statistical parsimony and Bayesian clustering analyses (Fig 4) exhibit different colour morphs that partially correspond to named forms. Specimens from Arizona and California were black with little colouration corresponding to the form *graefi*, described as lacking the typical yellow bands, which has been described as the principal form found in the west [25]. Samples from the southern group have distinctive yellow bands along the body, possibly representing either the nominate *exitiosa* or the form *barnesii*, while those from the “eastern” group also exhibit yellow stripes, although to a lesser extent. The single female sample in the eastern group is *edwardsii*, evident by the distinct orange ring on the fourth and fifth segments of the abdomen [25].

### Carmenta mimuli, Synanthedon decipiens, Synanthedon sapygaeformis, and Zenodoxus rubens

*Carmenta mimuli* showed a surprising amount of variation in the three populations sampled, with two lineages identified although with no geographical correlation, possibly representing multiple invasions from a diverse Mexican population, multiple southern refugia with subsequent admixture, or the presence of cryptic species. The two lineages identified are sympatric, and there are no described morphological variants in this species. Slight colour differences do not correspond to the lineages seen here. Extensive sampling of this species, as well as inclusion of nuclear markers, is required to identify whether these are in fact cryptic species. *Synanthedon decipiens* also showed clear divergence, with no shared haplotypes between the two southern locations sampled. As the divergence between the populations is minimal (and therefore likely more recent), perhaps these populations target different host plant species as the Sonoran scrub oak *Quercus turbinella* only extends as far east as Texas, while other *Quercus* (Fagaceae) species occur in Louisiana [72].

*Zenodoxus rubens* possessed multiple distinct lineages suggesting either multiple colonisation events into the southern United States, possibly as peripheral populations of a broader Meso-American species, or the presence of cryptic species. While little is known about Neotropical sesiids, two *Zenodoxus* species are known to occur in Baja California [78] and seven species have been described from the southwestern U.S. [25]. The samples studied here are consistent with the nominate form of *Z*. *rubens* as described by Eichlin and Duckworth [25], with the exception that the single divergent Texas sample has a hyaline region on the hindwings, identifying it as the *bexari* form. The divergences among groups range from 1.7% between the two Arizona groups and 3.3% between one Arizona group and the *bexari* Texas sample; distances such as the latter may suggest a separate species, or may be a result of missing intermediate samples. The lack of obvious morphological differences in the majority of samples suggests that the large divergences between the Arizona and Texas samples may represent a continuous cline of variation and limited dispersal capabilities. The presence of the *bexari* sample as the most distinct lineage raises additional questions; further sampling, particularly of *bexari* colour forms, is required to confirm its species status. The other species restricted to the southern states, *Synanthedon sapygaeformis*, may also represent a northern extension of a Meso-American species, or it may be endemic to the extreme south-east of the United States. *S*. *sapygaeformis* exhibited much higher variability than other southern species despite limited structure, possibly reflecting an older population. Eichlin and Duckworth [25] describe two colour forms for this species: the nominate form and the variant *floridensis*. All specimens examined here are of the more common *floridensis* form, suggesting that the differences in colour forms are not responsible for the variation.

## Conclusions

Despite the lack of targeted taxonomic sampling, the COI sequences in BOLD provided an excellent overview of the population genetic structure in North American sesiid moths. A number of species showed distinct genetic lineages, likely representing the signature of isolation during glacial periods as well as limited ongoing gene flow. For example, *Synanthedon acerni* and *S*. *exitiosa* each showed four distinct lineages, suggesting historical separation followed by low levels of contemporary gene flow. In contrast, the widespread *Pennisetia marginata* exhibited a starburst pattern and almost complete lack of variation despite samples from both eastern and western locations, indicating a single glacial refuge and recent bottleneck and/or origin.

The results show that despite similar life history traits, this group of insects shows highly variable evolutionary histories. Future work should focus not only on species of agricultural importance, such as *Synanthedon exitiosa*, but also on those of evolutionary interest highlighted here, such as *Albuna pyramidalis*, *Synanthedon acerni*, and *Zenodoxus rubens*. In all of these species, additional samples targeting potential physical barriers and putative refugia will help to clarify their evolutionary history. In addition, by incorporating additional markers it will be possible to clarify whether the variation seen here reflects diversity that arose in previously isolated populations or the presence of cryptic species.

## Supporting information

S1 Table Sample information for the 558 sesiid moths and outgroup castniid. The BOLD sample ID, GenBank Accession Number, Barcode Index Number (BIN), sampling location, and institute storing are given. GPS coordinates for the eight selected species are given where available.
(PDF)
