# Supplementary material for: A survey of molecular diversity and population genetic structure in North American clearwing moths (Lepidoptera: Sesiidae) using cytochrome c oxidase I
Source: PLoS One. 2018 Aug 22;13(8):e0202281. doi: 10.1371/journal.pone.0202281 (PMC6104984; doi:10.1371/journal.pone.0202281)
Supplement: S1 Table — The BOLD sample ID, GenBank Accession Number, Barcode Index Number (BIN), sampling location, and institute storing are given. GPS coordinates for the eight selected species are given where available. (PDF) [file pone.0202281.s001.pdf]

Supplementary Table 1. Sample information for the 558 sesiid moths and outgroup castniid. The BOLD sample ID, GenBank Accession Number (GenBank), Barcode Index Number (BIN), sampling location, and institute storing (Inst) are given. GPS coordinates for the eight selected species are given where available. Inst abbreviations are given below the table.

Species Name	BOLD ID	GenBank	BIN	Country	Province/ State	Inst	Lat	Long
Xanthocastnia evalthe	LTOLB430-09	HM377853	AAK0165	Guyana		FMNH		
Albuna fraxini	BIOUG25029-E12	n/a	AAZ1305	Canada	Ontario	CNC		
Albuna fraxini	CNCLEP00100591	n/a	AAZ1305	Canada	Quebec	CNC		
Albuna fraxini	CCDB-04612 D05	n/a	AAZ1305	United States	Michigan	FP		
Albuna fraxini	CCDB-04613 G05	n/a	AAZ1305	United States	Michigan	FP		
Albuna fraxini	CCDB-04613 G08	n/a	AAZ1305	United States	Michigan	HR		
Albuna fraxini	CCDB-19578-D10	n/a	AAZ1305	United States	Michigan	NMNH		
Albuna pyramidalis	UASM99834	HM902915	AAB5656	Canada	Alberta	UAB	52.72	-116.27
Albuna pyramidalis	08BBLEP-02366	KM552816	AAB5656	Canada	Alberta	BIO	49.08	-113.97
Albuna pyramidalis	08BBLEP-04746	KM553742	AAB5656	Canada	Alberta	BIO	51.20	-115.49
Albuna pyramidalis	08BBLEP-04780	HM377773	AAB5656	Canada	Alberta	BIO	49.07	-114.00
Albuna pyramidalis	08BBLEP-04781	HM377774	AAB5656	Canada	Alberta	BIO	49.07	-114.00
Albuna pyramidalis	08BBLEP-04782	HM377775	AAB5656	Canada	Alberta	BIO	49.07	-114.00
Albuna pyramidalis	CCDB-02111 F11	HM392874	AAB5656	Canada	British Columbia	FP	50.13	-122.96
Albuna pyramidalis	10BBCLP-0221	JF841260	AAB5656	Canada	British Columbia	BIO	51.44	-116.54
Albuna pyramidalis	10BBCLP-0222	JF841261	AAB5656	Canada	British Columbia	BIO	51.44	-116.54
Albuna pyramidalis	10BBCLP-0223	JF841262	AAB5656	Canada	British Columbia	BIO	51.44	-116.54
Albuna pyramidalis	AVBC 210-10	JF852647	AAB5656	Canada	British Columbia	BIO	49.30	-124.78
Albuna pyramidalis	AVBC 1128-11	KT147155	AAB5656	Canada	British Columbia	BIO	49.13	-124.70
Albuna pyramidalis	AVBC 1149-11	KT141609	AAB5656	Canada	British Columbia	BIO	50.08	-122.98
Albuna pyramidalis	BIOUG03547-E07	KM540048	AAB5656	Canada	Manitoba	BIO	50.68	-99.90
Albuna pyramidalis	04HBL003147	GU096624	AAB5656	Canada	Manitoba	BIO	58.73	-93.82
Albuna pyramidalis	04HBL003526	GU096625	AAB5656	Canada	Manitoba	CNC	58.73	-93.82
Albuna pyramidalis	04HBL003527	GU096623	AAB5656	Canada	Manitoba	BIO	58.73	-93.82
Albuna pyramidalis	07PROBE-00154	KT128506	AAB5656	Canada	Manitoba	CNC	58.62	-93.81

Albuna pyramidalis	07PROBE-00169	KT148052	AAB5656	Canada	Manitoba	CNC	58.62	-93.81
Albuna pyramidalis	07PROBE-00604	KT135424	AAB5656	Canada	Manitoba	BIO	58.63	-93.82
Albuna pyramidalis	07PROBE-10092	KT141652	AAB5656	Canada	Manitoba	CNC	58.63	-93.82
Albuna pyramidalis	07PROBE-10212	KT129168	AAB5656	Canada	Manitoba	CNC	58.74	-93.82
Albuna pyramidalis	09BBELE-1277	HM415091	AAB5656	Canada	Newfoundland	BIO	48.51	-53.95
Albuna pyramidalis	09BBELE-1278	HM415092	AAB5656	Canada	Newfoundland	BIO	48.51	-53.95
Albuna pyramidalis	09BBELE-1881	HM415495	AAB5656	Canada	Newfoundland	BIO	48.60	-53.94
Albuna pyramidalis	10BBCLP-0224	JF841263	AAB5656	Canada	Saskatchewan	BIO	53.59	-106.05
Albuna pyramidalis	KENWR 7183	n/a	AAB5656	United States	Alaska	BIO	66.70	-150.88
Albuna pyramidalis	KENWR TE-03	n/a	AAB5656	United States	Alaska	BIO	66.70	-150.88
Albuna pyramidalis	UAM:Ento:83460	KU874045	AAB5656	United States	Alaska	UAK	64.90	-147.53
Alcathoe verrugo	CCDB-19579-C08	n/a	ACC4086	United States	California	NMNH		
Carmenta anthracipennis	BIOUG26008-B05	n/a	AAM0561	Canada	Ontario	BIO		
Carmenta anthracipennis	UASM99829	HM902932	AAM0561	Canada	Saskatchewan	UAB		
Carmenta anthracipennis	CCDB-04612 A09	n/a	AAM0561	United States	Colorado	MSU		
Carmenta anthracipennis	CNCLEP 92341	n/a	AAM0561	United States	Louisiana	CNC		
Carmenta apache	CCDB-04613 F10	JF848556	AAQ1175	United States	Arizona	FP		
Carmenta apache	CCDB-04613 F12	n/a	AAQ1175	United States	Arizona	HR		
Carmenta arizonae	TAMUICEGR-0244	HQ977591	AAO0561	United States	Texas	TAMU		
Carmenta auritincta	CCDB-02114 A01	n/a	ABA8559	United States	Arizona	FP		
Carmenta auritincta	CCDB-02114 A02	HM392636	AAK2780	United States	Arizona	FP		
Carmenta auritincta	W. TAFT Lep 00075	HQ564747	AAK2780	United States	Arizona	HR		
Carmenta auritincta	W. TAFT Lep 00076	HQ564748	AAK2780	United States	Arizona	HR		
Carmenta auritincta	W. TAFT Lep 00077	HQ564749	AAK2780	United States	Arizona	HR		
Carmenta auritincta	W. TAFT Lep 00078	HQ564750	AAK2780	United States	Arizona	HR		
Carmenta auritincta	W. TAFT Lep 00079	HQ564751	AAK2780	United States	Arizona	HR		
Carmenta auritincta	W. TAFT Lep 00080	HQ564752	AAK2780	United States	Arizona	HR		
Carmenta bassiformis	CNCLEP 92340	n/a	ACD9133	United States	Louisiana	CNC		
Carmenta bassiformis	CCDB-04612 A02	n/a	AAY9855	United States	Pennsylvania	MSU		
Carmenta bassiformis	CCDB-04612 A03	n/a	AAY9855	United States	Pennsylvania	MSU		
Carmenta bassiformis	CCDB-04612 B06	n/a	AAY9856	United States	Pennsylvania	MSU		
Carmenta bassiformis	CCDB-19578-H07	n/a	AAY9855	United States	Pennsylvania	NMNH		

Carmenta bassiformis	BGS03483	n/a	AAW4297	United States	Tennessee	CCH		
Carmenta corni	CCDB-19578-H08	n/a	ACC3885	Canada	New Brunswick	NMNH		
Carmenta corni	CCDB-19578-H09	n/a	ACC3885	Canada	New Brunswick	NMNH		
Carmenta corni	CCDB-19578-H10	n/a	ACC3885	Canada	New Brunswick	NMNH		
Carmenta corni	CNCLEP00103231	n/a	ACC3885	Canada	Ontario	CNC		
Carmenta corni	CNCLEP00103232	n/a	ACC3885	Canada	Ontario	CNC		
Carmenta engelhardti	CCDB-19579-A02	n/a	ACC3943	United States	Arizona	NMNH		
Carmenta engelhardti	TAMUICEGR-0245	HQ977592	AAO0562	United States	Texas	TAMU		
Carmenta flaschkai	FP Lep 00628	n/a	ABU7383	United States	Texas	FP		
Carmenta giliae	CCDB-19579-A03	n/a	ACC3973	United States	Arizona	NMNH		
Carmenta giliae	CCDB-19579-A04	n/a	ACC3973	United States	Arizona	NMNH		
Carmenta giliae	CCDB-19579-A05	n/a	ACC3973	United States	Arizona	NMNH		
Carmenta giliae	CCDB-04612 A04	n/a	AAZ1402	United States	Wyoming	MSU		
Carmenta laurelae	CCDB-14649 C07	n/a	ABX1301	United States	Florida	MSU		
Carmenta laurelae	CCDB-14649 C09	n/a	ABX1301	United States	Florida	MSU		
Carmenta mariona	CCDB-04612 A05	n/a	AAZ1405	United States	New Mexico	MSU		
Carmenta mimuli	CSU-CPG-LEP001049	GU685605	AAD6727	United States	Arizona	CSU	31.49	-110.69
Carmenta mimuli	CSU-CPG-LEP001051	GU685599	AAD6727	United States	Arizona	CSU	31.49	-110.69
Carmenta mimuli	CSU-CPG-LEP001053	GU685601	AAD6727	United States	Arizona	CSU	31.49	-110.69
Carmenta mimuli	W. TAFT Lep 00041	HQ564720	AAD6727	United States	Arizona	HR	31.49	-110.69
Carmenta mimuli	W. TAFT Lep 00042	HQ564721	AAD6727	United States	Arizona	HR	31.49	-110.69
Carmenta mimuli	W. TAFT Lep 00043	HQ564722	AAD6727	United States	Arizona	FP	31.49	-110.69
Carmenta mimuli	W. TAFT Lep 00044	HQ564723	AAD6727	United States	Arizona	HR	31.49	-110.69
Carmenta mimuli	W. TAFT Lep 00045	HQ564724	AAD6727	United States	Arizona	FP	31.49	-110.69
Carmenta mimuli	W. TAFT Lep 00046	HQ564725	AAD6727	United States	Arizona	HR	31.49	-110.69
Carmenta mimuli	W. TAFT Lep 00048	HQ564727	AAD6727	United States	Arizona	HR	31.49	-110.69
Carmenta mimuli	W. TAFT Lep 00049	HQ564728	AAD6727	United States	Arizona	FP	31.49	-110.69
Carmenta mimuli	W. TAFT Lep 00050	HQ564729	AAD6727	United States	Arizona	FP	31.49	-110.69
Carmenta mimuli	W. TAFT Lep 00051	HQ564730	AAD6727	United States	Arizona	HR	32.33	-110.70
Carmenta mimuli	CNCLEP 92227	n/a	AAD6727	United States	Louisiana	CNC	31.54	-93.12
Carmenta mimuli	TAMUICEGR-0246	HQ977593	AAD6727	United States	Texas	TAMU	n/a	n/a
Carmenta mimuli	TAMUICEGR-0247	HQ977594	AAD6727	United States	Texas	TAMU	n/a	n/a

Carmenta mimuli	TAMUICEGR-0248	HQ977595	AAD6727	United States	Texas	TAMU	n/a	n/a
Carmenta odda	TAMUICEGR-0249	HQ977596	AAN9569	United States	Texas	TAMU		
Carmenta pallene	CCDB-04612 A06	n/a	AAZ1403	United States	Arizona	MSU		
Carmenta phoradendri	CCDB-19579-A09	n/a	ACC3952	United States	Texas	NMNH		
Carmenta phoradendri	CCDB-19579-A10	n/a	ACC3952	United States	Texas	NMNH		
Carmenta prosopis	CCDB-04612 A07	n/a	AAZ1404	United States	Arizona	MSU		
Carmenta prosopis	CCDB-14649 D05	n/a	AAZ1404	United States	Arizona	MSU		
Carmenta pyralidiformis	FP Lep 00571	HQ564784	AAM4361	United States	Louisiana	FP		
Carmenta pyralidiformis	FP Lep 00572	HQ564785	AAM4361	United States	Louisiana	FP		
Carmenta pyralidiformis	FP Lep 00574	HQ564787	AAM4361	United States	Louisiana	FP		
Carmenta pyralidiformis	CNCLEP 92222	n/a	AAM4361	United States	Louisiana	CNC		
Carmenta pyralidiformis	CCDB-19579-A11	n/a	AAM4361	United States	Maryland	NMNH		
Carmenta pyralidiformis	CCDB-04612 A08	n/a	AAM4361	United States	Michigan	MSU		
Carmenta querci	CCDB-04611 D01	n/a	ABW1603	United States	Arizona	HR		
Carmenta suffusata	CCDB-14649 E11	n/a	ABY1592	United States	Florida	MSU		
Carmenta tecta	CCDB-04612 D06	n/a	AAQ1177	United States	Arizona	FP		
Carmenta tecta	CCDB-04613 F07	JF848554	AAQ1177	United States	Arizona	FP		
Carmenta texana	CCDB-19579-A06	n/a	ACC3814	United States	Florida	NMNH		
Carmenta texana	CNCLEP00103233	n/a	ACC3814	United States	Georgia	CNC		
Carmenta texana	CNCLEP00103234	n/a	ACC3814	United States	Georgia	CNC		
Carmenta texana	CNCLEP00103236	n/a	ACC3814	United States	Georgia	CNC		
Carmenta verecunda	CSU-CPG-LEP001056	HM372884	AAK2801	United States	Wyoming	CSU		
Carmenta welchelorum	CCDB-19579-B06	n/a	ACC3779	United States	Texas	NMNH		
Carmenta welchelorum	CCDB-19579-B07	n/a	ACC3779	United States	Texas	NMNH		
Carmenta wellerae	W. TAFT Lep 00052	HQ564731	AAM3413	United States	Arizona	FP		
Carmenta wellerae	W. TAFT Lep 00053	HQ564732	AAM3413	United States	Arizona	HR		
Carmenta wellerae	W. TAFT Lep 00054	HQ564733	AAM3413	United States	Arizona	HR		
Carmenta wellerae	W. TAFT Lep 00055	HQ564734	AAM3413	United States	Arizona	HR		
Carmenta wellerae	W. TAFT Lep 00056	HQ564735	AAM3413	United States	Arizona	HR		
Carmenta wielgusi	CNCLEP00100928	n/a	ACG6005	United States	Arizona	CNC		
Carmenta wielgusi	CNCLEP00100929	n/a	ACG6005	United States	Arizona	CNC		
Cissuvora ampelopsis	CSU-CPG-LEP001065	GU685590	AAC1035	United States	Texas	CSU		

Cissuvora ampelopsis	CSU-CPG-LEP001066	GU685591	AAC1035	United States	Texas	CSU
Cissuvora ampelopsis	CSU-CPG-LEP001067	GU685584	AAC1035	United States	Texas	CSU
Cissuvora ampelopsis	CSU-CPG-LEP001068	GU685585	AAC1035	United States	Texas	CSU
Cissuvora ampelopsis	CSU-CPG-LEP001069	GU685586	AAC1035	United States	Texas	CSU
Cissuvora ampelopsis	HLC-27391	GU691547	AAC1035	United States	Texas	BIO
Cissuvora ampelopsis	HLC-27392	GU691557	AAC1035	United States	Texas	BIO
Cissuvora ampelopsis	HLC-27393	GU691558	AAC1035	United States	Texas	BIO
Cissuvora ampelopsis	HLC-27394	GU691559	AAC1035	United States	Texas	BIO
Cissuvora ampelopsis	HLC-27395	GU691560	AAC1035	United States	Texas	BIO
Cissuvora ampelopsis	HLC-27397	GU691546	AAC1035	United States	Texas	BIO
Cissuvora ampelopsis	HLC-27398	GU691554	AAC1035	United States	Texas	BIO
Cissuvora ampelopsis	HLC-27399	GU691555	AAC1035	United States	Texas	BIO
Cissuvora ampelopsis	HLC-27396	GU691544	AAC1035	United States	Texas	BIO
Euhagena emphytiformis	CCDB-14649 C06	n/a	ABX7874	United States	Arizona	MSU
Euhagena nebraskae	CCDB-14649 C08	n/a	ABX7873	United States	Colorado	MSU
Euhagena nebraskae	CCDB-19578-E01	n/a	ABX7873	United States	Colorado	NMNH
Euhagena nebraskae	CCDB-19578-E02	n/a	ABX7873	United States	Colorado	NMNH
Euhagena nebraskae	CCDB-19578-E03	n/a	ABX7873	United States	Colorado	NMNH
Hymenoclea palmii	CSU-CPG-LEP001063	GU685588	AAE5101	United States	Arizona	CSU
Hymenoclea palmii	CSU-CPG-LEP001064	GU685589	AAE5101	United States	Arizona	CSU
Hymenoclea palmii	W. TAFT Lep 00074	HQ564746	AAE5101	United States	Arizona	FP
Hymenoclea palmii	TAMUICEGR-0253	HQ977600	AAN3146	United States	Texas	TAMU
Melittia calabaza	CCDB-19578-B05	n/a	ACF2431	United States	Arizona	NMNH
Melittia calabaza	CCDB-19578-B06	n/a	ABY7076	United States	Texas	NMNH
Melittia calabaza	TAMUICEGR-0223	HQ977572	ABY7076	United States	Texas	TAMU
Melittia cucurbitae	CNCLEP00100592	n/a	AAK7015	Canada	Ontario	CNC
Melittia cucurbitae	CNCLEP00121549	n/a	AAK7015	Canada	Quebec	CNC
Melittia cucurbitae	TPF-94-1106	n/a	AAK7015	United States	Maryland	UMD
Melittia cucurbitae	CCDB-19578-B08	n/a	AAK7015	United States	Maryland	NMNH
Melittia cucurbitae	CCDB-19578-B09	n/a	ACF2087	United States	Virginia	NMNH
Melittia cucurbitae	CCDB-19578-B10	n/a	ACF2087	United States	Virginia	NMNH
Melittia gloriosa	CCDB-04613 G04	JF848559	AAH3520	United States	Arizona	HR

Melittia gloriosa	CCDB-19578-B11	n/a	AAH3520	United States	Arizona	NMNH
Melittia gloriosa	CCDB-19578-B12	n/a	AAH3520	United States	Arizona	NMNH
Melittia gloriosa	W. TAFT Lep 00073	HQ564745	AAM4219	United States	Arizona	HR
Melittia gloriosa	CSU-CPG-LEP001018	GU685633	AAH3520	United States	Colorado	CSU
Melittia gloriosa	CSU-CPG-LEP001019	GU685626	AAH3520	United States	Oklahoma	CSU
Melittia grandis	BIOUG01830-E05	n/a	AAZ1355	United States	Arizona	BIO
Melittia grandis	CCDB-04612 A10	n/a	AAZ1355	United States	Arizona	MSU
Melittia grandis	CNCLEP00104832	n/a	AAZ1355	United States	Texas	CNC
Melittia grandis	CNCLEP00104833	n/a	AAZ1355	United States	Texas	CNC
Melittia grandis	CCDB-19578-C02	n/a	AAZ1355	United States	Texas	NMNH
Melittia snowii	CCDB-04612 E03	n/a	AAF4357	United States	Arizona	MSU
Melittia snowii	W. TAFT Lep 00071	HQ564744	AAF4357	United States	Arizona	HR
Melittia snowii	CSU-CPG-LEP001016	GU685631	AAF4357	United States	Nebraska	CSU
Melittia snowii	CSU-CPG-LEP001017	GU685632	AAF4357	United States	Texas	CSU
Melittia snowii	CCDB-19578-B07	n/a	AAF4357	United States	Texas	NMNH
Melittia snowii	TAMUICEGR-0224	HQ977573	AAF4357	United States	Texas	TAMU
Osminia donahueorum	CCDB-04612 A12	n/a	AAY9965	United States	Arizona	MSU
Osminia donahueorum	CCDB-04612 B07	n/a	AAY9965	United States	Arizona	MSU
Osminia donahueorum	CCDB-04612 B08	n/a	AAY9965	United States	Arizona	MSU
Osminia donahueorum	CCDB-19578-A12	n/a	AAY9965	United States	Arizona	NMNH
Osminia donahueorum	CCDB-19578-B01	n/a	AAY9965	United States	Arizona	NMNH
Osminia ruficornis	CCDB-04612 A11	n/a	AAZ1372	United States	Arizona	MSU
Osminia ruficornis	CCDB-14649 D12	n/a	AAZ1372	United States	Arizona	MSU
Osminia ruficornis	CCDB-14649 E04	n/a	AAZ1372	United States	Arizona	MSU
Osminia ruficornis	CCDB-14649 E06	n/a	AAZ1372	United States	Arizona	MSU
Palmia praecedens	CCDB-04612 E06	n/a	AAN1534	United States	Arizona	HR
Palmia praecedens	CCDB-04612 E08	n/a	AAN1534	United States	Arizona	FP
Palmia praecedens	W. TAFT Lep 00065	HQ564740	AAN1534	United States	Arizona	FP
Palmia praecedens	CCDB-14649 C11	n/a	AAN1534	United States	Arizona	MSU
Palmia praecedens	CCDB-14649 D02	n/a	AAN1534	United States	Arizona	MSU
Palmia praecedens	CCDB-14649 D04	n/a	AAN1534	United States	Arizona	MSU
Palmia praecedens	CCDB-14649 D06	n/a	AAN1534	United States	Arizona	MSU

Paranthrene asilipennis	CNCLEP00104841	n/a	AAN1855	United States	Georgia	CNC		
Paranthrene asilipennis	CCDB-04610 D02	n/a	AAN1855	United States	Georgia	HR		
Paranthrene asilipennis	W. TAFT Lep 00007	HQ564693	AAN1855	United States	Michigan	FP		
Paranthrene dollii	CCDB-04612 E04	n/a	AAM5081	United States	Michigan	HR		
Paranthrene dollii	CCDB-04612 G07	n/a	AAM5081	United States	Michigan	HR		
Paranthrene dollii	CCDB-19578-C12	n/a	ACC3979	United States	Michigan	NMNH		
Paranthrene fenestrata	CCDB-04612 B01	n/a	ABU6848	United States	Arizona	MSU		
Paranthrene fenestrata	CCDB-04612 B02	n/a	ABU6848	United States	Arizona	MSU		
Paranthrene fenestrata	CCDB-14649 C03	n/a	ABU6848	United States	Arizona	MSU		
Paranthrene fenestrata	CCDB-14649 F03	n/a	ABU6848	United States	Arizona	MSU		
Paranthrene fenestrata	CCDB-19578-D02	n/a	ABU6848	United States	Arizona	NMNH		
Paranthrene fenestrata	CCDB-19578-D03	n/a	ABU6848	United States	Arizona	NMNH		
Paranthrene robiniae	UASM77894	n/a	AAL3386	Canada	Alberta	GP		
Paranthrene robiniae	CCDB-04612 G06	n/a	AAY9980	Canada	British Columbia	FP		
Paranthrene robiniae	CCDB-19578-D06	n/a	ACC4022	United States	California	NMNH		
Paranthrene robiniae	CCDB-19578-D07	n/a	ACC4022	United States	California	NMNH		
Paranthrene robiniae	CCDB-19578-D08	n/a	ACC4022	United States	California	NMNH		
Paranthrene simulans	W. TAFT Lep 00061	HQ564736	AAM2835	United States	Arizona	HR		
Paranthrene simulans	W. TAFT Lep 00062	HQ564737	AAM2835	United States	Arizona	HR		
Paranthrene simulans	W. TAFT Lep 00063	HQ564738	AAM2835	United States	Arizona	HR		
Paranthrene simulans	W. TAFT Lep 00064	HQ564739	ABY4995	United States	Arizona	HR		
Paranthrene simulans	CCDB-04613 F06	JF848553	AAM2835	United States	Michigan	FP		
Paranthrene simulans	DNA-ATBI-2752	GU089135	AAI6314	United States	Tennessee	BIO		
Paranthrene tabaniformis	CNCLEP00104844	n/a	ACH1653	Canada	Manitoba	CNC		
Pennisetia marginata	10BBCLP-0217	JF841256	AAC5621	Canada	Alberta	BIO	53.67	-112.87
Pennisetia marginata	10BBCLP-0218	JF841257	AAC5621	Canada	Alberta	BIO	53.62	-112.88
Pennisetia marginata	10BBCLP-0219	JF841258	AAC5621	Canada	Alberta	BIO	53.62	-112.88
Pennisetia marginata	10BBCLP-0220	JF841259	AAC5621	Canada	Alberta	BIO	53.63	-112.86
Pennisetia marginata	10BBCLP-0226	JF841265	AAC5621	Canada	Alberta	BIO	53.69	-112.81
Pennisetia marginata	10BBCLP-0227	JF841266	AAC5621	Canada	Alberta	BIO	53.63	-112.86
Pennisetia marginata	CNCLEP00076691	JN286312	AAC5621	Canada	British Columbia	CNC	49.06	-122.47
Pennisetia marginata	CNCLEP00076692	JN286313	AAC5621	Canada	British Columbia	CNC	49.06	-122.47

Pennisetia marginata	CNCLEP00076693	JN286314	AAC5621	Canada	British Columbia	CNC	49.06	-122.47
Pennisetia marginata	09BBELE-0159	GU690379	AAC5621	Canada	New Brunswick	BIO	46.90	-60.78
Pennisetia marginata	09BBELE-1051	HM436293	AAC5621	Canada	New Brunswick	BIO	45.55	-65.02
Pennisetia marginata	09BBELE-1052	HM436294	AAC5621	Canada	New Brunswick	BIO	45.55	-65.02
Pennisetia marginata	09BBELE-1055	HM436297	AAC5621	Canada	New Brunswick	BIO	45.55	-65.02
Pennisetia marginata	09BBELE-1116	HM436358	AAC5621	Canada	New Brunswick	BIO	45.66	-65.02
Pennisetia marginata	09BBELE-1117	HM436359	AAC5621	Canada	New Brunswick	BIO	45.66	-65.02
Pennisetia marginata	09BBELE-1118	HM436360	AAC5621	Canada	New Brunswick	BIO	45.66	-65.02
Pennisetia marginata	09BBELE-1119	HM436361	AAC5621	Canada	New Brunswick	BIO	45.66	-65.02
Pennisetia marginata	09BBELE-1120	HM436362	AAC5621	Canada	New Brunswick	BIO	45.66	-65.02
Pennisetia marginata	09BBELE-1484	HM415293	AAC5621	Canada	New Brunswick	BIO	45.66	-65.14
Pennisetia marginata	BIOUG08430-A08	KT129736	AAC5621	Canada	New Brunswick	BIO	44.62	-63.57
Pennisetia marginata	BIOUG10063-H10	KR450577	AAC5621	Canada	Ontario	BIO	44.85	-79.87
Pennisetia marginata	OMAFRA06-160	n/a	AAC5621	Canada	Ontario	BIO	n/a	n/a
Pennisetia marginata	BIOUG06751-A07	n/a	AAC5621	Canada	Ontario	AS	45.51	-76.98
Pennisetia marginata	BIOUG10669-C04	KR450511	AAC5621	Canada	Quebec	BIO	48.86	-64.38
Pennisetia marginata	BIOUG10669-C05	KR454256	AAC5621	Canada	Quebec	BIO	48.86	-64.38
Pennisetia marginata	BIOUG10669-C06	KR447757	AAC5621	Canada	Quebec	BIO	48.86	-64.38
Pennisetia marginata	BIOUG12164-F02	KR454074	AAC5621	Canada	Quebec	BIO	48.86	-64.38
Pennisetia marginata	BIOUG12164-F03	KR452491	AAC5621	Canada	Quebec	BIO	48.86	-64.38
Pennisetia marginata	2009GM-0173	n/a	AAC5621	United States	California	BIO	36.88	-121.63
Pennisetia marginata	CNCLEP00076694	JN286315	AAC5621	United States	Utah	CNC	41.83	-111.32
Pennisetia marginata	CNCLEP00076695	JN286316	AAC5621	United States	Utah	CNC	41.83	-111.32
Pennisetia marginata	CNCLEP00076696	JN286317	AAC5621	United States	Utah	CNC	41.83	-111.32
Pennisetia marginata	CNCLEP00076697	JN286318	AAC5621	United States	Utah	CNC	41.83	-111.32
Pennisetia marginata	CNCLEP00076698	JN286319	AAC5621	United States	Utah	CNC	41.83	-111.32
Pennisetia marginata	CNCLEP00076699	JN286320	AAC5621	United States	Utah	CNC	41.83	-111.32
Pennisetia marginata	CNCLEP00076700	JN286321	AAC5621	United States	Utah	CNC	41.83	-111.32
Penstemonia clarkei	CCDB-04613 G01	JF848557	AAQ0630	United States	Arizona	FP		
Penstemonia clarkei	CCDB-04613 G03	JF848558	AAQ0630	United States	Arizona	HR		
Penstemonia clarkei	CCDB-04612 B03	n/a	AAQ0630	United States	Arizona	MSU		
Penstemonia clarkei	CCDB-14649 B05	n/a	AAQ0630	United States	Arizona	MSU		
Penstemonia clarkei	CCDB-14649 B07	n/a	AAQ0630	United States	Arizona	MSU		

Penstemonia clarkei	CCDB-14649 B09	n/a	AAQ0630	United States	Arizona	MSU
Penstemonia dammersi	CCDB-19579-B08	n/a	ACC4033	United States	California	NMNH
Penstemonia dammersi	CCDB-19579-B09	n/a	ACC4033	United States	California	NMNH
Penstemonia edwardsii	CCDB-04612 E02	n/a	AAZ0346	United States	Arizona	FP
Penstemonia hennei	CCDB-19579-B11	n/a	ACC4072	United States	California	NMNH
Podosesia syringae	CNCLEP00041170	HQ965223	AAC0843	Canada	British Columbia	CNC
Podosesia syringae	BIOUG21233-A07	n/a	AAC0843	Canada	Ontario	CNC
Podosesia syringae	W. TAFT Lep 00036	n/a	AAC0843	United States	Michigan	HR
Podosesia syringae	DNA-ATBI-2753	GU089180	AAC0842	United States	Tennessee	BIO
Sannina uroceriformis	CNCLEP00100589	n/a	AAM4620	United States	Louisiana	CNC
Sannina uroceriformis	CNCLEP00100590	n/a	AAM4620	United States	Louisiana	CNC
Sannina uroceriformis	FP Lep 00561	HQ564774	AAM4620	United States	Louisiana	FP
Sannina uroceriformis	FP Lep 00563	HQ564776	AAM4620	United States	Louisiana	FP
Sannina uroceriformis	FP Lep 00565	HQ564778	AAM4620	United States	Louisiana	FP
Sesia apiformis	CNCLEP00024589	n/a	AAD4130	United States	New York	CNC
Sesia spartani	UASM99832	HM902916	AAL5811	Canada	Alberta	UAB
Sesia spartani	UASM99420	HM902917	AAL5811	Canada	Alberta	UAB
Sesia spartani	UASM99424	HM902918	AAL5811	Canada	Alberta	UAB
Sesia spartani	W. TAFT Lep 00008	HQ564694	AAL5811	United States	Michigan	HR
Sesia spartani	CCDB-04612 E01	n/a	AAL5811	United States	Michigan	HR
Sesia tibialis	UASM99831	n/a	AAC3589	Canada	Alberta	UAB
Sesia tibialis	BOX-2218 C08	HM376422	AAC3587	Canada	British Columbia	FP
Sesia tibialis	TAMUICEGR-0211	HQ977563	AAN2777	United States	Arizona	TAMU
Sesia tibialis	W. TAFT Lep 00067	HQ564741	AAC3587	United States	California	HR
Sesia tibialis	W. TAFT Lep 00068	HQ564742	AAC3587	United States	California	HR
Sesia tibialis	CSU-CPG-LEP001023	GU685653	AAC3588	United States	California	CSU
Sesia tibialis	CSU-CPG-LEP001002	HM372881	AAC3589	United States	Colorado	CSU
Sesia tibialis	CSU-CPG-LEP001024	GU685654	AAC3588	United States	Montana	CSU
Sesia tibialis	CSU-CPG-LEP001020	GU685627	AAC3588	United States	Utah	CSU
Sesia tibialis	CCDB-02114 A08	HM392641	AAC3588	United States	Washington	FP
Sesia tibialis	CCDB-02114 A09	HM392642	AAC3588	United States	Washington	FP
Sesia tibialis	CSU-CPG-LEP001001	GU685655	AAC3588	United States	Wyoming	CSU

Sesia tibialis	CSU-CPG-LEP001003	GU685652	AAC3588	United States	Wyoming	CSU		
Sesia tibialis	CSU-CPG-LEP001004	GU685641	AAC3588	United States	Wyoming	CSU		
Sophona greenfieldi	CSU-CPG-LEP001006	GU685643	AAD1388	United States	Arizona	CSU		
Sophona greenfieldi	CSU-CPG-LEP001007	GU685637	AAD1388	United States	Arizona	CSU		
Sophona greenfieldi	CSU-CPG-LEP001008	GU685638	AAD1388	United States	Arizona	CSU		
Sophona greenfieldi	CSU-CPG-LEP001010	GU685640	AAD1388	United States	Arizona	CSU		
Sophona greenfieldi	W. TAFT Lep 00037	HQ564716	AAD1388	United States	Arizona	HR		
Sophona greenfieldi	W. TAFT Lep 00038	HQ564717	AAD1388	United States	Arizona	HR		
Sophona greenfieldi	W. TAFT Lep 00039	HQ564718	AAD1388	United States	Arizona	HR		
Sophona greenfieldi	CCDB-02114 A03	HM902902	AAD1388	United States	Arizona	FP		
Sophona greenfieldi	CCDB-02114 A04	HM392637	AAD1388	United States	Arizona	FP		
Sophona snellingi	W. TAFT Lep 00011	HQ564697	ABX6078	United States	Arizona	FP		
Sophona snellingi	W. TAFT Lep 00012	n/a	ABX6078	United States	Arizona	HR		
Sophona snellingi	W. TAFT Lep 00013	HQ564698	ABX6078	United States	Arizona	HR		
Sophona snellingi	W. TAFT Lep 00015	HQ564700	ABX6078	United States	Arizona	HR		
Sophona snellingi	W. TAFT Lep 00017	n/a	ABX6078	United States	Arizona	HR		
Sophona snellingi	W. TAFT Lep 00018	HQ564701	ABX6078	United States	Arizona	FP		
Sophona snellingi	W. TAFT Lep 00019	HQ564702	ABX6078	United States	Arizona	HR		
Sophona snellingi	W. TAFT Lep 00020	HQ564703	ABX6078	United States	Arizona	HR		
Sophona snellingi	CNCLEP 84187	n/a	ABX6078	United States	Arizona	CNC		
Sophona snellingi	CNCLEP 84188	n/a	ABX6078	United States	Arizona	CNC		
Synanthedon acerni	MNBTT-2350	KT143149	ABZ2531	Canada	New Brunswick	BIO	45.59	-66.39
Synanthedon acerni	MNBTT-2351	KT125151	ABZ2531	Canada	New Brunswick	BIO	45.59	-66.39
Synanthedon acerni	MNBTT-2352	KT147970	ABZ2531	Canada	New Brunswick	BIO	45.59	-66.39
Synanthedon acerni	MNBTT-2353	KT145058	ABZ2531	Canada	New Brunswick	BIO	45.59	-66.39
Synanthedon acerni	MNBTT-2354	KT125836	ABZ2531	Canada	New Brunswick	BIO	45.59	-66.39
Synanthedon acerni	MNBTT-2355	KT142964	ABZ2531	Canada	New Brunswick	BIO	45.59	-66.39
Synanthedon acerni	MNBTT-2356	KT135274	ABZ2531	Canada	New Brunswick	BIO	45.55	-66.38
Synanthedon acerni	MNBTT-2357	KT142887	ABZ2531	Canada	New Brunswick	BIO	45.35	-67.21
Synanthedon acerni	MNBTT-2358	KT147317	ABZ2531	Canada	New Brunswick	BIO	45.35	-67.21
Synanthedon acerni	MNBTT-2359	KT147898	ABZ2531	Canada	New Brunswick	BIO	45.35	-67.21
Synanthedon acerni	MNBTT-2360	KT145407	ABZ2531	Canada	New Brunswick	BIO	45.35	-67.21
Synanthedon acerni	MNBTT-2361	KT125411	ABZ2531	Canada	New Brunswick	BIO	45.35	-67.21

Synanthedon acerni	09BBELE-2518	HM416109	ABZ2531	Canada	Newfoundland	BIO	48.65	-53.92
Synanthedon acerni	09BBELE-2525	HM416115	ABZ2531	Canada	Newfoundland	BIO	48.65	-53.92
Synanthedon acerni	09BBELE-2554	HM416143	ABZ2531	Canada	Newfoundland	BIO	48.65	-53.92
Synanthedon acerni	09BBELE-2559	HM416148	ABZ2531	Canada	Newfoundland	BIO	48.65	-53.92
Synanthedon acerni	10BBCLP-0666	JF841695	AAA8523	Canada	Ontario	BIO	41.93	-82.51
Synanthedon acerni	PPBP-2046	KT138615	AAA8523	Canada	Ontario	BIO	44.38	-80.57
Synanthedon acerni	04HBL005514	GU094329	AAA8523	Canada	Ontario	BIO	43.54	-80.13
Synanthedon acerni	2005-ONT-344	GU092148	AAA8523	Canada	Ontario	BIO	43.54	-80.13
Synanthedon acerni	0102-ONT-0022	KT133157	AAA8523	Canada	Ontario	BIO	43.54	-80.13
Synanthedon acerni	0102-ONT-0023	KT135193	AAA8523	Canada	Ontario	BIO	43.54	-80.13
Synanthedon acerni	0102-ONT-0024	KT144391	AAA8523	Canada	Ontario	BIO	43.54	-80.13
Synanthedon acerni	2006-ONT-0649	KT137825	AAA8523	Canada	Ontario	BIO	43.54	-80.13
Synanthedon acerni	jflandry2519	n/a	AAA8523	Canada	Quebec	CNC	45.53	-76.00
Synanthedon acerni	jflandry2520	n/a	AAA8523	Canada	Quebec	CNC	45.53	-76.00
Synanthedon acerni	jflandry2521	n/a	AAA8523	Canada	Quebec	CNC	45.53	-76.00
Synanthedon acerni	jflandry2522	n/a	AAA8523	Canada	Quebec	CNC	45.53	-76.00
Synanthedon acerni	jflandry2523	n/a	AAA8523	Canada	Quebec	CNC	45.53	-76.00
Synanthedon acerni	jflandry2601	n/a	AAA8523	Canada	Quebec	CNC	46.09	-74.28
Synanthedon acerni	MDH000055	n/a	AAA8523	Canada	Quebec	DH	45.53	-75.99
Synanthedon acerni	MDH001564	n/a	AAA8523	Canada	Quebec	DH	45.47	-73.08
Synanthedon acerni	MDH002017	n/a	AAA8523	Canada	Quebec	DH	46.59	-73.20
Synanthedon acerni	05-CTATBI-0286	n/a	AAA8523	United States	Connecticut	BIO	41.76	-72.65
Synanthedon acerni	CNCLEP00025809	n/a	AAA8524	United States	Florida	CNC	27.19	-81.34
Synanthedon acerni	06-JKA-0380	n/a	ABZ2531	United States	Georgia	BIO	34.42	-85.11
Synanthedon acerni	06-JKA-0532	n/a	ABZ2531	United States	Georgia	BIO	34.42	-85.10
Synanthedon acerni	06-JKA-0533	n/a	ABZ2531	United States	Georgia	BIO	34.42	-85.10
Synanthedon acerni	SNS10IL-00136	n/a	AAA8523	United States	Illinois	BIO	38.23	-88.19
Synanthedon acerni	SNS10IL-00151	n/a	AAA8523	United States	Illinois	BIO	38.23	-88.19
Synanthedon acerni	FP Lep 00564	HQ564777	ACF5513	United States	Louisiana	FP	30.52	-89.99
Synanthedon acerni	09BBLEP-04526	GU694264	AAA8523	United States	Michigan	BIO	43.53	-86.11
Synanthedon acerni	09BBLEP-04527	GU694265	AAA8523	United States	Michigan	BIO	43.53	-86.11
Synanthedon acerni	09BBLEP-04698	GU694390	AAA8523	United States	Michigan	BIO	43.53	-86.11
Synanthedon acerni	CCDB-19578-D12	n/a	ACF5513	United States	Mississippi	NMNH	n/a	n/a

Synanthedon acerni	TAMUICEGR-0226	HQ977575	AAA8523	United States	Texas	TAMU	n/a	n/a
Synanthedon acerni	CWM-96-0562	n/a	ABZ2531	United States	West Virginia	UMD	38.88	-78.87
Synanthedon acerrubri	BIOUG22868-E12	n/a	AAE2739	Canada	Ontario	BIO		
Synanthedon acerrubri	2005-ONT-241	GU092149	AAE2739	Canada	Ontario	BIO		
Synanthedon acerrubri	BIOUG22868-E11	n/a	AAE2739	Canada	Ontario	BIO		
Synanthedon acerrubri	jflandry0021	GU096122	AAE2739	Canada	Quebec	CNC		
Synanthedon acerrubri	jflandry0738	GU096123	AAE2739	Canada	Quebec	CNC		
Synanthedon acerrubri	CCDB-04613 F09	JF848555	AAE2739	United States	Georgia	FP		
Synanthedon acerrubri	CCDB-02114 A07	HM392640	AAE2739	United States	Michigan	FP		
Synanthedon acerrubri	DNA-ATBI-2772	GU089275	AAE2739	United States	Tennessee	BIO		
Synanthedon albicornis	TAMUICEGR-0225	HQ977574	AAN9927	United States	California	TAMU		
Synanthedon alleri	CCDB-19578-E09	n/a	ACC3976	United States	Maryland	NMNH		
Synanthedon alleri	CCDB-14649 B02	n/a	ACE6125	United States	South Carolina	MSU		
Synanthedon alleri	CCDB-14649 B04	n/a	ACE6125	United States	South Carolina	MSU		
Synanthedon arctica	04HBL003148	GU097106	AAI0243	Canada	Manitoba	CNC		
Synanthedon arctica	04HBL003149	GU097105	AAI0243	Canada	Manitoba	CNC		
Synanthedon arctica	BIOUG17055-F04	KT143925	AAI0243	Canada	Northwest Territories	BIO		
Synanthedon arkansasensis	CCDB-19578-F02	n/a	ACC5027	United States	Arkansas	NMNH		
Synanthedon arkansasensis	06-JKA-0587	n/a	AAK1072	United States	Georgia	BIO		
Synanthedon bibionipennis	UASM99827	HM902929	AAE2729	Canada	British Columbia	UAB		
Synanthedon bibionipennis	AVBC 1369-12	KT147142	AAE2729	Canada	British Columbia	BIO		
Synanthedon bibionipennis	08BBLEP-00775	KM543068	AAE2729	Canada	Saskatchewan	BIO		
Synanthedon bibionipennis	08BBLEP-00776	KM548723	AAE2729	Canada	Saskatchewan	BIO		
Synanthedon bibionipennis	08BBLEP-01759	KM548881	AAE2729	Canada	Saskatchewan	BIO		
Synanthedon bibionipennis	CSU-CPG-LEP001054	GU685602	AAE2729	United States	Wyoming	CSU		
Synanthedon bibionipennis	CSU-CPG-LEP001055	GU685596	AAE2729	United States	Wyoming	CSU		
Synanthedon bolteri	UASM99817	HM902923	AAL9504	Canada	Alberta	UAB		
Synanthedon bolteri	UASM99824	HM902924	AAL9504	Canada	Alberta	UAB		
Synanthedon bolteri	10BBCLP-0225	JF841264	AAL9504	Canada	Alberta	BIO		
Synanthedon bolteri	BIOUG21006-D10	n/a	AAL9504	Canada	Ontario	BIO		
Synanthedon bolteri	CCDB-04613 F11	n/a	AAL9504	United States	Michigan	FP		
Synanthedon canadensis	CCDB-14649 B03	n/a	ABX1263	Canada	British Columbia	MSU		

Synanthedon canadensis	CNCLEP00100593	n/a	ABX1263	Canada	British Columbia	CNC		
Synanthedon canadensis	CNCLEP00100594	n/a	ABX1263	Canada	British Columbia	CNC		
Synanthedon canadensis	CNCLEP00100595	n/a	ABX1263	Canada	British Columbia	CNC		
Synanthedon culiciformis	CNCLEP00100931	n/a	ACC4829	Canada	British Columbia	CNC		
Synanthedon culiciformis	CNCLEP00100932	n/a	ACC4829	Canada	British Columbia	CNC		
Synanthedon culiciformis	CCDB-19578-E04	n/a	ACC4829	United States	Oregon	NMNH		
Synanthedon decipiens	FP Lep 00569	HQ564782	AAH5191	United States	Louisiana	FP	30.52	-89.99
Synanthedon decipiens	FP Lep 00573	HQ564786	AAH5191	United States	Louisiana	FP	30.52	-89.99
Synanthedon decipiens	FP Lep 00575	HQ564788	AAH5191	United States	Louisiana	FP	30.52	-89.99
Synanthedon decipiens	CNCLEP 92228	n/a	AAH5191	United States	Louisiana	CNC	30.52	-89.95
Synanthedon decipiens	CNCLEP 92229	n/a	AAH5191	United States	Louisiana	CNC	30.52	-89.95
Synanthedon decipiens	HLC-27400	GU691556	AAH5191	United States	Texas	BIO	n/a	n/a
Synanthedon decipiens	HLC-27401	GU691543	AAH5191	United States	Texas	BIO	n/a	n/a
Synanthedon decipiens	TAMUICEGR-0227	HQ977576	AAH5191	United States	Texas	TAMU	n/a	n/a
Synanthedon decipiens	TAMUICEGR-0229	HQ977578	AAH5191	United States	Texas	TAMU	n/a	n/a
Synanthedon decipiens	TAMUICEGR-0230	HQ977579	AAH5191	United States	Texas	TAMU	n/a	n/a
Synanthedon exitiosa	W. TAFT Lep 00021	HQ564704	ACE6293	United States	Arizona	HR	32.37	-110.70
Synanthedon exitiosa	W. TAFT Lep 00022	HQ564705	ACE6293	United States	Arizona	HR	32.37	-110.70
Synanthedon exitiosa	W. TAFT Lep 00023	HQ564706	ACE6293	United States	Arizona	HR	32.37	-110.70
Synanthedon exitiosa	W. TAFT Lep 00024	HQ564707	ACE6293	United States	Arizona	HR	32.37	-110.70
Synanthedon exitiosa	W. TAFT Lep 00025	HQ564708	ACE6293	United States	Arizona	HR	32.37	-110.70
Synanthedon exitiosa	W. TAFT Lep 00026	HQ564709	ACE6293	United States	Arizona	HR	32.37	-110.70
Synanthedon exitiosa	TAMUICEGR-0231	HQ977580	ABZ1050	United States	Arkansas	TAMU	n/a	n/a
Synanthedon exitiosa	TAMUICEGR-0232	HQ977581	ABZ1050	United States	Arkansas	TAMU	n/a	n/a
Synanthedon exitiosa	TAMUICEGR-0233	HQ977582	ABZ1050	United States	Arkansas	TAMU	n/a	n/a
Synanthedon exitiosa	JLB-0034	n/a	AAC1774	United States	California	JB	n/a	n/a
Synanthedon exitiosa	JLB-0068	n/a	AAC1774	United States	California	JB	n/a	n/a
Synanthedon exitiosa	JLB-0071	n/a	AAC1774	United States	California	JB	n/a	n/a
Synanthedon exitiosa	CSU-CPG-LEP001027	GU685622	ABZ1050	United States	Colorado	CSU	39.59	-108.11
Synanthedon exitiosa	CSU-CPG-LEP001028	GU685623	ABZ1050	United States	Colorado	CSU	39.59	-108.11
Synanthedon exitiosa	CSU-CPG-LEP001031	GU685618	ACE6293	United States	Colorado	CSU	39.59	-108.11
Synanthedon exitiosa	FP Lep 00556	HQ564769	ABZ1050	United States	Louisiana	FP	30.52	-89.99
Synanthedon exitiosa	BIOUG02920-F12	n/a	ABZ1050	United States	Oklahoma	BIO	36.74	-95.95

Synanthedon exitiosa	BIOUG02920-H05	n/a	ABZ1050	United States	Oklahoma	BIO	36.74	-95.95
Synanthedon exitiosa	BIOUG21233-A06	n/a	AAC1773	Canada	Ontario	CNC	n/a	n/a
Synanthedon exitiosa	BIOUG24423-B06	n/a	AAC1773	Canada	Ontario	CNC	n/a	n/a
Synanthedon exitiosa	BIOUG25029-F01	n/a	AAC1773	Canada	Ontario	CNC	n/a	n/a
Synanthedon exitiosa	BIOUG25029-F02	n/a	AAC1773	Canada	Ontario	CNC	n/a	n/a
Synanthedon exitiosa	BIOUG25029-F03	n/a	AAC1773	Canada	Ontario	CNC	n/a	n/a
Synanthedon exitiosa	OMAFRA06-006	n/a	AAC1773	Canada	Ontario	CNC	n/a	n/a
Synanthedon exitiosa	CSU-CPG-LEP001029	GU685624	AAC1773	United States	Colorado	CSU	n/a	n/a
Synanthedon exitiosa	CSU-CPG-LEP001030	GU685625	AAC1773	United States	Colorado	CSU	n/a	n/a
Synanthedon exitiosa	06-JKA-0240	n/a	AAC1773	United States	Georgia	BIO	32.60	-82.33
Synanthedon fatifera	CNCLEP 00113944	n/a	AAM3114	United States	Louisiana	CNC		
Synanthedon fatifera	CNCLEP 00113945	n/a	AAM3114	United States	Louisiana	CNC		
Synanthedon fatifera	FP Lep 00552	HQ564767	AAM3114	United States	Louisiana	FP		
Synanthedon fatifera	FP Lep 00554	HQ564768	AAM3114	United States	Louisiana	FP		
Synanthedon fatifera	CNCLEP 92230	n/a	AAM3114	United States	Louisiana	CNC		
Synanthedon fatifera	CNCLEP 92231	n/a	AAM3114	United States	Louisiana	CNC		
Synanthedon fatifera	CNCLEP 92232	n/a	AAM3114	United States	Louisiana	CNC		
Synanthedon fulvipes	CNCLEP00103240	n/a	AAN0121	Canada	Ontario	CNC		
Synanthedon fulvipes	CNCLEP00103241	n/a	AAN0121	Canada	Ontario	CNC		
Synanthedon fulvipes	CNCLEP00103245	n/a	AAN0121	Canada	Quebec	CNC		
Synanthedon fulvipes	W. TAFT Lep 00002	HQ564692	AAN0121	United States	Michigan	HR		
Synanthedon fulvipes	CCDB-04613 G06	JF848560	AAN0121	United States	Michigan	HR		
Synanthedon geliformis	CNCLEP00026125	n/a	AAF2831	United States	Florida	CNC		
Synanthedon geliformis	CNCLEP00026126	n/a	AAF2831	United States	Florida	CNC		
Synanthedon geliformis	CNCLEP00026127	n/a	AAF2831	United States	Florida	CNC		
Synanthedon kathyae	FP Lep 00546	HQ564762	AAM3731	United States	Louisiana	FP		
Synanthedon kathyae	FP Lep 00558	HQ564771	AAM3731	United States	Louisiana	FP		
Synanthedon kathyae	FP Lep 00560	HQ564773	AAM3731	United States	Louisiana	FP		
Synanthedon kathyae	FP Lep 00562	HQ564775	AAM3731	United States	Louisiana	FP		
Synanthedon kathyae	TAMUICEGR-0234	HQ977583	AAM3731	United States	Texas	TAMU		
Synanthedon kathyae	TAMUICEGR-0235	HQ977584	AAM3731	United States	Texas	TAMU		
Synanthedon mellinipennis	UASM99825	HM902930	AAL5967	Canada	British Columbia	UAB		

Synanthedon mellinipennis	UASM99826	n/a	AAL5967	Canada	British Columbia	UAB
Synanthedon mellinipennis	UASM99828	HM902931	AAL5967	Canada	British Columbia	UAB
Synanthedon myopaeformis	jflandry2542	n/a	AAC4688	Canada	British Columbia	CNC
Synanthedon myopaeformis	jflandry2543	n/a	AAC4688	Canada	British Columbia	CNC
Synanthedon myopaeformis	jflandry2571	n/a	AAC4688	Canada	British Columbia	CNC
Synanthedon myopaeformis	CNCLEP00027819	n/a	AAC4688	Canada	British Columbia	CNC
Synanthedon myopaeformis	CNCLEP00027820	n/a	AAC4688	Canada	British Columbia	CNC
Synanthedon myopaeformis	CNCLEP00027821	n/a	AAC4688	Canada	British Columbia	CNC
Synanthedon myopaeformis	CNCLEP00027822	n/a	AAC4688	Canada	British Columbia	CNC
Synanthedon novaroensis	UASM57518	n/a	AAD5330	Canada	Alberta	CNC
Synanthedon novaroensis	UASM57581	n/a	AAD5330	Canada	Alberta	CNC
Synanthedon novaroensis	BOX-2219 A04	GU661886	AAD5330	Canada	British Columbia	FP
Synanthedon novaroensis	CCDB-04613 G09	JF848561	AAD5330	United States	Alaska	HR
Synanthedon novaroensis	CSU-CPG-LEP001032	GU685619	ABZ1363	United States	California	CSU
Synanthedon novaroensis	CSU-CPG-LEP001033	GU685620	ABZ1363	United States	California	CSU
Synanthedon novaroensis	CSU-CPG-LEP001034	GU685621	ABZ1363	United States	California	CSU
Synanthedon novaroensis	CSU-CPG-LEP001035	GU685614	ABZ1363	United States	California	CSU
Synanthedon pictipes	BIOUG12513-E09	KR446903	AAK1487	Canada	Newfoundland	BIO
Synanthedon pictipes	CNCLEP 94300	n/a	AAK1487	United States	Louisiana	CNC
Synanthedon pictipes	CCDB-04612 D08	n/a	AAK1487	United States	Pennsylvania	HR
Synanthedon pictipes	CCDB-04612 D10	n/a	AAK1487	United States	Pennsylvania	HR
Synanthedon pini	CNCLEP00082534	JN279256	AAV3629	Canada	Ontario	CNC
Synanthedon pini	W. TAFT Lep 00006	n/a	AAV3629	United States	Michigan	FP
Synanthedon pini	CCDB-14649 C10	n/a	AAV3629	United States	Michigan	MSU
Synanthedon pini	CCDB-14649 C12	n/a	AAV3629	United States	Michigan	MSU
Synanthedon pini	CCDB-14649 D01	n/a	AAV3629	United States	Michigan	MSU
Synanthedon polygoni	CSU-CPG-LEP001038	GU685617	AAD7381	United States	California	CSU
Synanthedon polygoni	CSU-CPG-LEP001039	GU685610	AAD7381	United States	California	CSU
Synanthedon proxima	CCDB-04613 F01	n/a	ABA7149	United States	Michigan	FP
Synanthedon refulgens	CNCLEP 92226	n/a	ABY0693	United States	Louisiana	CNC
Synanthedon refulgens	CCDB-14649 D10	n/a	ABY0693	United States	Michigan	MSU
Synanthedon resplendens	CCDB-19578-G03	n/a	ACC4858	United States	California	NMNH

Synanthedon resplendens	CCDB-19578-G04	n/a	ACC4858	United States	California	NMNH		
Synanthedon resplendens	CCDB-19578-G05	n/a	ACC4858	United States	California	NMNH		
Synanthedon rhododendri	CCDB-04643 H06	HQ957068	AAO9584	United States	Georgia	FP		
Synanthedon rhododendri	CCDB-14649 D11	n/a	AAO9584	United States	Pennsylvania	MSU		
Synanthedon rhododendri	CCDB-14649 E01	n/a	AAO9584	United States	Pennsylvania	MSU		
Synanthedon rhododendri	CCDB-14649 E03	n/a	AAO9584	United States	Pennsylvania	MSU		
Synanthedon richardsi	CCDB-19578-G06	n/a	ACC4942	United States	Georgia	NMNH		
Synanthedon rileyana	CCDB-19578-G09	n/a	ABA8701	United States	Arkansas	NMNH		
Synanthedon rileyana	CCDB-19578-G11	n/a	ABA8701	United States	Georgia	NMNH		
Synanthedon rileyana	CCDB-19578-G10	n/a	ABA8701	United States	Kentucky	NMNH		
Synanthedon rileyana	W. TAFT Lep 00003	n/a	ABA8701	United States	Michigan	HR		
Synanthedon rileyana	CCDB-04613 F04	n/a	ABA8701	United States	Michigan	HR		
Synanthedon rubrofascia	FP Lep 00568	HQ564781	AAM3212	United States	Louisiana	FP		
Synanthedon rubrofascia	FP Lep 00570	HQ564783	AAM3212	United States	Louisiana	FP		
Synanthedon rubrofascia	DNA-ATBI-0707	GU090207	AAE8739	United States	North Carolina	n/a		
Synanthedon rubrofascia	DNA-ATBI-0708	GU090206	AAE8739	United States	North Carolina	n/a		
Synanthedon rubrofascia	DNA-ATBI-0099	GU090205	AAE8739	United States	Tennessee	n/a		
Synanthedon rubrofascia	DNA-ATBI-0100	GU090204	AAE8739	United States	Tennessee	n/a		
Synanthedon rubrofascia	TAMUICEGR-0239	HQ977586	AAM3212	United States	Texas	TAMU		
Synanthedon rubrofascia	TAMUICEGR-0240	HQ977587	AAM3212	United States	Texas	TAMU		
Synanthedon sapygaeformis	CNCLEP00025953	n/a	AAB9434	United States	Florida	CNC	27.17	-81.35
Synanthedon sapygaeformis	CNCLEP00026119	n/a	AAB9434	United States	Florida	CNC	27.19	-81.34
Synanthedon sapygaeformis	CNCLEP00026120	n/a	AAB9434	United States	Florida	CNC	27.19	-81.34
Synanthedon sapygaeformis	CNCLEP00026121	n/a	AAB9434	United States	Florida	CNC	27.19	-81.34
Synanthedon sapygaeformis	CNCLEP00026122	n/a	AAB9434	United States	Florida	CNC	27.19	-81.34
Synanthedon sapygaeformis	CNCLEP00026123	n/a	AAB9434	United States	Florida	CNC	27.19	-81.34
Synanthedon sapygaeformis	CNCLEP00026124	n/a	AAB9434	United States	Florida	CNC	27.19	-81.34
Synanthedon sapygaeformis	CNCLEP00026131	n/a	AAB9434	United States	Florida	CNC	27.19	-81.34
Synanthedon sapygaeformis	CNCLEP00026132	n/a	AAB9434	United States	Florida	CNC	27.19	-81.34
Synanthedon sapygaeformis	CNCLEP00026133	n/a	AAB9434	United States	Florida	CNC	27.19	-81.34
Synanthedon sapygaeformis	CNCLEP00026134	n/a	AAB9434	United States	Florida	CNC	27.19	-81.34
Synanthedon sapygaeformis	CNCLEP00026135	n/a	AAB9434	United States	Florida	CNC	27.19	-81.34

Synanthedon sapygaeformis	CNCLEP00026136	n/a	AAB9434	United States	Florida	CNC	27.19	-81.34
Synanthedon sapygaeformis	CNCNoctuoidea12800	n/a	AAB9434	United States	Florida	CNC	27.28	-82.13
Synanthedon sapygaeformis	CNCNoctuoidea12801	n/a	AAB9434	United States	Florida	CNC	27.28	-82.13
Synanthedon sapygaeformis	CNCNoctuoidea12802	n/a	AAB9434	United States	Florida	CNC	27.28	-82.13
Synanthedon sapygaeformis	TAMUICEGR-0241	HQ977588	AAB9434	United States	Texas	TAMU	n/a	n/a
Synanthedon saxifragae	UASM58419	HM902927	AAM0008	Canada	Alberta	UAB		
Synanthedon scitula	SNS10IL-00992	n/a	ABA2389	United States	Illinois	BIO		
Synanthedon scitula	SNS10IL-01026	n/a	ABA2389	United States	Illinois	BIO		
Synanthedon scitula	FP Lep 00566	HQ564779	AAF2956	United States	Louisiana	FP		
Synanthedon scitula	FP Lep 00567	HQ564780	ACF0446	United States	Louisiana	FP		
Synanthedon scitula	CNCLEP00057839	GU679059	AAF2956	United States	Louisiana	CNC		
Synanthedon scitula	CNCLEP00057840	GU679060	ACF0446	United States	Louisiana	CNC		
Synanthedon scitula	TAMUICEGR-0242	HQ977589	ACF5052	United States	Texas	TAMU		
Synanthedon sequoiae	CNCLEP00104863	n/a	ABX1237	Canada	British Columbia	CNC		
Synanthedon sequoiae	CCDB-22973-A02	n/a	ABX1237	Canada	British Columbia	CFS		
Synanthedon sequoiae	CCDB-14649 E07	n/a	ABX1237	United States	California	MSU		
Synanthedon sequoiae	CCDB-14649 E09	n/a	ABX1237	United States	California	MSU		
Synanthedon sigmoidea	CCDB-04613 F05	JF848552	AAN0120	United States	Michigan	HR		
Synanthedon sigmoidea	CCDB-14649 E08	n/a	AAN0120	United States	Michigan	MSU		
Synanthedon sigmoidea	CCDB-14649 E10	n/a	AAN0120	United States	Michigan	MSU		
Synanthedon sigmoidea	CCDB-14649 E12	n/a	AAN0120	United States	Michigan	MSU		
Synanthedon sigmoidea	CCDB-14649 F01	n/a	AAN0120	United States	Michigan	MSU		
Synanthedon tipuliformis	UASM99806	HM902919	AAC1840	Canada	Alberta	UAB		
Synanthedon tipuliformis	UASM99805	HM902920	AAC1840	Canada	Alberta	UAB		
Synanthedon viburni	BIOUG20646-A09	KR940874	AAN0117	Canada	Ontario	BIO		
Vitacea admiranda	CCDB-04612 B04	n/a	ABA8665	United States	Oklahoma	MSU		
Vitacea admiranda	CCDB-14649 B01	n/a	ABA8665	United States	Texas	MSU		
Vitacea admiranda	CCDB-14649 D03	n/a	ABA8665	United States	Texas	MSU		
Vitacea admiranda	TAMUICEGR-0218	HQ977569	ABA8665	United States	Texas	TAMU		
Vitacea admiranda	TAMUICEGR-0219	HQ977570	ABA8665	United States	Texas	TAMU		
Vitacea polistiformis	FP Lep 00557	HQ564770	AAM2795	United States	Louisiana	FP		
Vitacea polistiformis	FP Lep 00559	HQ564772	AAM2795	United States	Louisiana	FP		

Vitacea polistiformis	DNA-ATBI-0709	GU090234	AAD9368	United States	North Carolina	n/a		
Vitacea polistiformis	DNA-ATBI-0101	GU090233	AAD9368	United States	Tennessee	n/a		
Vitacea polistiformis	DNA-ATBI-0102	GU090232	AAD9368	United States	Tennessee	n/a		
Vitacea polistiformis	TAMUICEGR-0220	HQ977571	AAM2795	United States	Texas	TAMU		
Vitacea polistiformis	CB-07-1002	n/a	AAD9369	United States	Virginia	UMD		
Vitacea scepsiformis	CNCLEP00026128	n/a	AAF3813	United States	Florida	CNC		
Vitacea scepsiformis	CNCLEP00026129	n/a	AAF3813	United States	Florida	CNC		
Vitacea scepsiformis	CNCLEP00026130	n/a	AAF3814	United States	Florida	CNC		
Zenodoxus canescens	CCDB-14649 B06	n/a	ABY1206	United States	Colorado	MSU		
Zenodoxus palmii	W. TAFT Lep 00033	HQ564713	AAN0270	United States	Arizona	FP		
Zenodoxus rubens	W. TAFT Lep 00029	HQ564710	AAH8327	United States	Arizona	HR	31.88	-110.66
Zenodoxus rubens	W. TAFT Lep 00032	HQ564712	AAH8327	United States	Arizona	FP	31.88	-110.66
Zenodoxus rubens	CCDB-02113 H06	HM392631	AAH8327	United States	Arizona	FP	31.88	-110.66
Zenodoxus rubens	CCDB-02113 H07	HM392632	ACF1524	United States	Arizona	FP	31.88	-110.66
Zenodoxus rubens	TAMUICEGR-0213	HQ977564	AAN3016	United States	Texas	TAMU	n/a	n/a
Zenodoxus rubens	TAMUICEGR-0214	HQ977565	AAN3017	United States	Texas	TAMU	n/a	n/a
Zenodoxus rubens	TAMUICEGR-0215	HQ977566	AAN3017	United States	Texas	TAMU	n/a	n/a
Zenodoxus rubens	TAMUICEGR-0216	HQ977567	AAN3017	United States	Texas	TAMU	n/a	n/a

Institutions

BIO – Biodiversity Institute of Ontario
CFS – Canadian Forest Service, Northern Forestry Centre
CNC – Canadian National Collection of Insects, Arachnids and Nematodes
CCH – College of Charleston
CSU – Colorado State University, C.P. Gillette Museum of Arthropod Diversity
FMNH – Florida Museum of Natural History
MSU – Michigan State University Museum
NMNH – Smithsonian Institution National Museum of Natural History
TAMU – Texas A&M University
UAK – University of Alaska Museum
UAB – University of Alberta, Strickland Museum of Entomology
UMD – University of Maryland

Private Collectors

AS – Alex Smith
DH – Daniel Handfield
FP – Franz Pühringer
GP – Gregory Pohl
HR – Hans Riefenstahl
JB – Jennifer Bundy
